# Time to Pregnancy: A Computational Method for Using the Duration of Non-Conception for Predicting Conception

**DOI:** 10.1371/journal.pone.0046544

**Published:** 2012-10-04

**Authors:** Peter D. Sozou, Geraldine M. Hartshorne

**Affiliations:** 1 Warwick Medical School, University of Warwick, Coventry, United Kingdom; 2 Faculty of Business and Economics, RWTH Aachen University, Aachen, Germany; 3 Centre for Philosophy of Natural and Social Science, London School of Economics and Political Science, London, United Kingdom; 4 Centre for Reproductive Medicine, University Hospitals Coventry and Warwickshire NHS Trust, Coventry, United Kingdom; University of East Piedmont, Italy

## Abstract

An important problem in reproductive medicine is deciding when people who have failed to become pregnant without medical assistance should begin investigation and treatment. This study describes a computational approach to determining what can be deduced about a couple's future chances of pregnancy from the number of menstrual cycles over which they have been trying to conceive. The starting point is that a couple's fertility is inherently uncertain. This uncertainty is modelled as a probability distribution for the chance of conceiving in each menstrual cycle. We have developed a general numerical computational method, which uses Bayes' theorem to generate a posterior distribution for a couple's chance of conceiving in each cycle, conditional on the number of previous cycles of attempted conception. When various metrics of a couple's expected chances of pregnancy were computed as a function of the number of cycles over which they had been trying to conceive, we found good fits to observed data on time to pregnancy for different populations. The commonly-used standard of 12 cycles of non-conception as an indicator of subfertility was found to be reasonably robust, though a larger or smaller number of cycles may be more appropriate depending on the population from which a couple is drawn and the precise subfertility metric which is most relevant, for example the probability of conception in the next cycle or the next 12 cycles. We have also applied our computational method to model the impact of female reproductive ageing. Results indicate that, for women over the age of 35, it may be appropriate to start investigation and treatment more quickly than for younger women. Ignoring reproductive decline during the period of attempted conception added up to two cycles to the computed number of cycles before reaching a metric of subfertility.

## Introduction

Among infertile couples, a longer period of time without conception is associated with a lower probability of conception in the following six or twelve months [1], [2]. In considering what period of non-conception should precede investigation and fertility treatment, there is a trade-off [3], [4]. On the one hand, there are costs associated with investigating, and perhaps treating, couples who may otherwise have conceived without medical assistance [5], [6]. On the other hand, delaying the treatment of infertile couples may result in worse outcomes. Those affected may have children later than intended (with the increased risks and costs associated with delayed parenting) or fewer children than they would have wished, or may even lose the chance to have their own genetic child. In this study, we consider what information can be derived from a given period of non-conception, and how this information may be used to determine the optimal timing of investigation and treatment. While the process of attempted conception over time can be modelled as occurring in continuous time [7], for our analysis it is more convenient to model the process as one that occurs over successive discrete menstrual cycles.

There are two reasons why couples who are trying to conceive experience differing outcomes with respect to when - and if - conception occurs. The first is pure randomness. Even if all such couples were identical in every relevant respect, becoming pregnant is an inherently stochastic process. It can be compared to repeatedly casting a die until a six is achieved: success may occur the first time, or the second, and so on; but over any finite number of attempts it may not be achieved at all.

The second reason why outcomes differ is that couples are not identical: they vary in characteristics which have a bearing on the probability of achieving a pregnancy in a new cycle. In simple terms, some couples can be said to be more fertile than others [8], [9]. This variation among couples, however, does not remove the component of randomness in the conception process. Suppose, for example, that couple A is more fertile than couple B in some objective way, while couple B is not completely sterile. Then, over a fixed number of cycles, couple A is more likely to conceive than couple B, but it remains possible that couple B will conceive and couple A will not. Similarly, one usually needs fewer tosses of a fair coin to achieve a head than throws of a fair die to achieve a six, but achieving a six before a head would not be a particularly surprising occurrence.

Overall variation in outcomes results from a combination of both randomness and variation among couples in their underlying fertility [10], [11]. Given some cohort of couples trying for a baby, it will tend to be the more fertile couples who conceive sooner and the less fertile who conceive later or not at all [12], [13], though the effects of chance mean that some couples with lower fertility will conceive earlier than other couples with higher fertility. It follows that those couples who fail to achieve a conception within a given number of cycles will *tend to be* those of lower fertility, but there will nevertheless be some higher fertility couples among their number. As the number of cycles increases, the population of couples who have not yet achieved a conception will become increasing biased towards the lower end of the fertility spectrum, which is the main reason why the probability of conception declines after a number of cycles of non-conception [1], [2].

Empirical models to predict a couple's chance of conceiving spontaneously are discussed by Hunault *et al.* [14], who propose the use of a prediction method based on duration of non-conception, female age, previous fertility status (i.e. ‘primary infertility’ vs ‘secondary infertility’), percentage of motile sperm, and whether referral is by a general practitioner or a gynaecologist. Good prediction results from this method have been reported [15]. The key point is that duration of non-conception is an important component of prediction methods for estimating the chance of conceiving spontaneously. This implies that variables other than duration of non-conception do not provide *perfect information* about a couple's fertility. In other words, there is some component of a couple's fertility that cannot be directly measured from variables such as age, physical characteristics, previous medical history, fertility history and currently available medical tests. This conclusion is consistent with the large proportion of patients whose failure to conceive is not explained by routine investigations; the UK Human Fertilisation and Embryology Authority reported that “Nearly a quarter of patients [in 2008] had unexplained infertility” [16].

A number of authors have described analytical methods for calculating conception probabilities over time for a couple whose probability of conception each cycle is drawn from a probability distribution (e.g. [11], [12], [17]–[21]). In this study, we have implemented this form of probabilistic analysis numerically, as a computer program. This allows a heterogeneous cohort of couples – or a single couple - to be followed through successive cycles of attempted conception, where conception is defined as achievement of a clinical pregnancy. The program can be applied to any distribution of probability of conception chosen by the user, and allows for a finite prior probability of sterility to be specified. For couples who fail to conceive, a posterior distribution of the monthly probability of conception is computed. Previous studies have calculated the probability of conception within a given period (e.g. the next 12 or 24 months) as a function of the number of months of attempted conception (e.g. [12], [19]). Our study calculates these and additional metrics of fertility, based on percentiles of the posterior distribution of the monthly probability of conception. These metrics are relevant to treatment decisions. The approach has potential applications in decision support systems for determining the best course of treatment and it supports the optimal use of resources, taking into account all the available information. We apply this analysis to specific examples of distributions for the probability of conception per cycle. We then apply our computational method to a specific population model in which couples experience declining fertility with age, i.e. a couple's monthly probability of conception decreases over time. This allows detailed modelling of the effect of female age on a couple's conception prospects over time and on the number of cycles of non-conception that would elapse before a chosen fertility metric exceeds a specified threshold indicating subfertility. It also allows computation of how declining fertility due to ageing during attempted conception influences the calculated number of cycles before a subfertility threshold is reached.

## Basic Analysis

### The concept of the intrinsic conception rate

Demographers use the term “fecundability” (e.g. [7], [8], [10]–[13], [17]–[21]) to refer to a couple's probability of conception per unit time or per cycle, while Cramer et al. [22] use the term “monthly fecundability”. However, it is necessary to distinguish between two different measures of this probability.

The first is the measure that would apply if one had perfect information about a couple, i.e. if there were no uncertainty in their fertility. We refer to this as the couple's *intrinsic conception rate*, defined as their probability of achieving a pregnancy in the next cycle, if they have not yet achieved a pregnancy. It corresponds to the *monthly fecundity rate* in [23]. It is not precisely measurable because current methods for fertility assessment give only imperfect information about a couple's fertility. Therefore, any estimate of a couple's intrinsic conception rate is subject to some uncertainty.

The second is a measure of a couple's probability of conception in the next cycle, according to the (generally imperfect) information available. This is the expected value of the intrinsic conception rate, reflecting uncertainty, and for this study will be simply referred to as the probability of conception.

In statistical terminology, the intrinsic conception rate represents the latent true value of the parameter, whereas what we refer to as the probability of conception is an estimate of this parameter under uncertainty.

In this study, we define conception as achievement of a clinical pregnancy. Not all clinical pregnancies lead to a live birth. Thus, the probability of a pregnancy leading to a live birth is on average about 10% lower than the probability of a clinical pregnancy per se. As a live birth is ultimately the desired outcome, it may appear that live birth would be the relevant end-point. There are two reasons why, for this study, it is preferable to use clinical pregnancy as the end-point. The first is consistency with the way pregnancy rates have been measured in previous studies (e.g. [1], [24]). The second is that a couple will generally be aware of a clinical pregnancy (even if it does not lead to live birth), and the prognosis for a couple who have suffered a failed pregnancy will generally be different from that of a couple who have never achieved a clinical pregnancy. However, it should be recognised that the intrinsic conception rate is not a perfect measure of reproductive health. Some women have a condition that leads to both rapid pregnancy and frequent miscarriage [25].

In the basic analysis, which we apply to four specific examples, it is assumed that the intrinsic conception rate for a given couple remains constant over the period of time being modelled. In practice there will be some decline in the intrinsic conception rate for a couple due to the effects of ageing. Is it reasonable to ignore this decline for modelling purposes? A rationale for so doing is that, where the female partner is not older than her mid 30 s [26], and does not have a reduced ovarian reserve [27], it seems likely that the systematic decline in a couple's fertility during the modelled time period when they are attempting to conceive will be relatively small [28]. Consider, for example, a couple in their 20 s who fail to conceive over 12 cycles. For prediction purposes, the probability of this couple conceiving on the 13^th^ cycle will be smaller than it was on their first cycle attempting to conceive [14], [15]. The main cause of this difference is that 12 cycles of non-conception are an *indicator* that this couple's intrinsic conception rate is low. In other words, the period of non-conception has yielded information about their intrinsic conception rate. The effect of the couple's intrinsic conception rate declining due to ageing over the time period in question (in this case about one year) is likely to be smaller. Conversely, in a couple where the woman is approaching her menopause, an additional year of age may make a much greater contribution to the declining intrinsic conception rate. It is of interest to have some idea of how much difference ignoring reproductive ageing during the time period of attempted conception makes to results. We have explored this by implementing a model of reproductive ageing, described in the section on reproductive ageing and applied in example 5 of this study. The implications for patient management decisions of reproductive ageing during the period of attempted conception are also discussed.

For the basic analysis, the assumption of a constant intrinsic conception rate also requires that a couple's successive cycles are statistically independent with respect to the probability of conception. This does not assume that all cycles are equally good. Suppose, for example, that a woman ovulates successfully in 50% of her cycles, and that whether or not she ovulates on a given cycle is independent of previous cycles; and that, given the quality of her partner's sperm, she has a 1/3 probability of conceiving in cycles when she ovulates, and a zero chance in cycles when she does not ovulate. Then her chance of becoming pregnant in each new cycle is ½×1/3 = 1/6: this is the intrinsic conception rate for her and her partner.

### Pregnancy likelihood for a given intrinsic conception rate

Suppose a couple has an intrinsic conception rate *y*. We assume that 0≤*y*<1, i.e. it is possible for a couple to be completely sterile but it is not possible for a couple to be so fertile that they are certain to achieve a pregnancy on the first cycle that they try. Let *P*(*y*,*n*) be the cumulative probability of conception for that couple, i.e. the probability that the couple will conceive within *n* cycles. This is the cumulative distribution function for a geometric random variable, given by
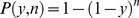
(1)Suppose for example that *y* = 0.2. Then the chance of the couple conceiving within two cycles (i.e. *n* = 2) is, from equation (1), 1−(1−0.2)^2^ = 0.36. Therefore the couple will have a 36% chance of conceiving within two cycles.

**Figure 1:**
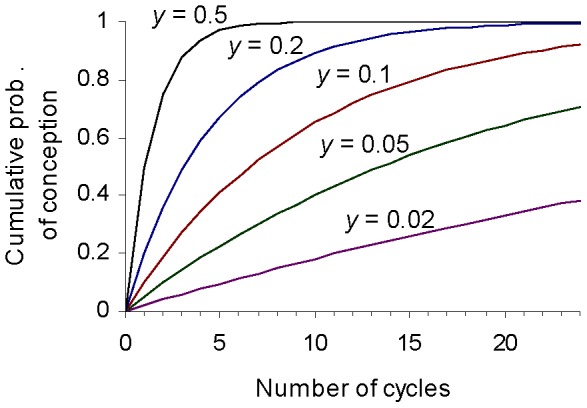
Plots showing the cumulative probability of conception P, i.e. the probability of conception within *n* cycles, for different values of the intrinsic conception rate *y*.

For any *y* and *n* it is possible to calculate *P*(*y*, *n*) using equation (1). Figure 1 shows how the probability of conception *P* within a given number of cycles depends on the number of cycles *n* and the intrinsic conception rate *y*.

It is possible to invert equation (1) to calculate the intrinsic conception rate *y* which will give a certain probability *P* of achieving a pregnancy within *n* cycles (see for example [22]). From equation (1) we obtain
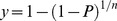
(2)Suppose, for example, that we wish to find the intrinsic conception rate *y* which corresponds to a probability of 0.5 of conceiving within 12 cycles. From equation (2), this value of *y* is 0.0561. That is, *P*(0.0561, 12) = 0.5. So a couple with an intrinsic conception rate of 5.61%, would have a 50% chance of achieving a pregnancy within 12 cycles. A couple with an intrinsic conception rate higher than 5.61% would have a >50% chance of conceiving within 12 cycles, and one with an intrinsic conception rate <5.61% would have a <50% chance of conceiving within 12 cycles.

Table 1 shows values of the intrinsic conception rate corresponding to probabilities of conception ranging from 0.05 (5%) to 0.95 (95%), within 12 or 24 cycles, as given by equation (2). The table illustrates just how large a role chance plays in determining whether or not a couple conceives. For example, 80% of couples with an intrinsic conception rate of 13.6% will conceive within 12 cycles, but 20% of couples with an intrinsic conception rate of 1.84% will also conceive within the same time period. Failure to conceive within a given number of cycles therefore serves as an *indicator* of a couple's intrinsic conception rate, but it is a noisy indicator. Numerical examples of cumulative conception probabilities are also given in [23], Table 1.

**Table 1:** Intrinsic conception rates corresponding to probability *P* of conceiving within 12 or 24 cycles.

A: 12 cycles:							
Probability *P* of conceiving within 12 cycles	0.05 (5%)	0.1 (10%)	0.2 (20%)	0.5 (50%)	0.8 (80%)	0.9 (90%)	0.95 (95%)
Corresponding intrinsic conception rate *y*	0.0043 (0.43%)	0.0087 (0.87%)	0.0184 (1.84%)	0.0561 (5.61%)	0.136 (13.6%)	0.175 (17.5%)	0.221 (22.1%)
B: 24 cycles:							
Probability *P* of conceiving within 24 cycles	0.05 (5%)	0.1 (10%)	0.2 (20%)	0.5 (50%)	0.8 (80%)	0.9 (90%)	0.95 (95%)
Corresponding intrinsic conception rate *y*	0.0021 (0.21%)	0.0044 (0.44%)	0.0093 (0.93%)	0.0285 (2.85%)	0.065 (6.5%)	0.101 (10.1%)	0.117 (11.7%)

### Dealing with a probability distribution for the intrinsic conception rate

As discussed above, a couple's true fertility is unknown. This is the case both when a couple starts trying to conceive, and when a couple seeks medical help because of a presumed fertility problem. We model this uncertainty by assuming a *probability distribution* for the intrinsic conception rate. One interpretation of the distribution applies where the couple are drawn at random from a specific population, and no additional information relevant to the couple's fertility is available. The probability distribution of the intrinsic conception rate then corresponds to the distribution of intrinsic conception rates within that population. This interpretation is appropriate for understanding the distribution of time-to-conception in a cohort of couples which is considered as a single population [1], [2]. Another interpretation applies to a couple defined by a specific set of objective attributes, or measurements: a prediction model may produce a point estimate of the chance of conception on the next cycle from these attributes, but the point estimate will be subject to uncertainty, with this uncertainty having an impact on the calculation of the probability of conception over several cycles. The probability distribution represents this uncertainty, with the distribution changing with the addition of more information about the couple. The probability distribution of the intrinsic conception rate for a specific couple can in this case be understood by treating the couple as being drawn from a *hypothetical* population of couples which share the same observed, objective characteristics but vary in certain hidden variables and therefore vary in their intrinsic conception rates.

For descriptive simplicity, we will treat the examples of distributions considered below as referring to populations.

We describe the probability distribution for a couple's intrinsic conception rate at the start of their attempt to achieve a pregnancy (i.e. after zero cycles), as the *prior distribution*. We can regard the couple as being drawn from a *prior population*. After one or more cycles of attempted conception, the information that a couple has failed to conceive can be used to update the prior distribution, leading to a new distribution which we describe as a *posterior distribution*.

As equation (1) implies and Figure 1 shows, a couple is more likely to conceive within some finite number of cycles if their intrinsic conception rate is higher. It follows that if a couple fails to conceive, it is relatively more likely that their intrinsic conception rate is towards the lower end of the distribution. Specifically, this means that the posterior distribution for the intrinsic conception rate, conditional on a couple not conceiving within some finite number of cycles, will exhibit a lower probability density than the prior distribution at the most fertile end of the distribution, and a higher probability density than the prior distribution at the least fertile end.

Bayes' theorem provides the formula for generating a posterior probability distribution from a prior distribution and an event which carries relevant information. In this case, the relevant event is that conception has not occurred.

Let *f*(*y*) be the prior distribution for a couple's intrinsic conception rate. We begin by calculating the probability *s*(*n*) that a couple drawn at random from the prior population will fail to conceive within *n* cycles. This constitutes a ‘survival function’, where ‘survival’ in this case means not conceiving within a given number of cycles. If the intrinsic conception rate *y* for this couple were known, the survival function would, by definition, be given by 1−*P*(*y*,*n*). From (1), this is (1−*y*)*^n^*. However, the value of *y* is unknown, so the survival function is derived by taking an appropriate weighted mean over all values of *y* in the distribution. The problem closely resembles that of survival under an uncertain hazard rate [29]. Letting s(n) be the probability that a couple drawn from the distribution *f*(*y*) have not conceived after n cycles, we obtain:
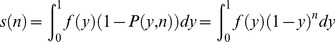
(3)with *s*(0) = 1 for any *f*(*y*).

Let *f_n_*(*y*) be the distribution of the intrinsic conception rate conditional on *n* cycles of non-conception. Applying Bayes' theorem for a continuous distribution [30], we obtain
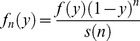
(4)We have written a computer program in C which, for any prior distribution of the intrinsic conception rate, numerically computes the probability of conception within any specified number of cycles, and the posterior distribution after any specified number of cycles of non-conception. The program also produces a number of metrics, as a function of the number of cycles of attempted conception, which are relevant to decisions about fertility assessment and treatment. These metrics are: the probability of the couple being sterile; percentiles of the (posterior) distribution of the intrinsic conception rate; and the probability of conceiving within a specified number of subsequent cycles. Details of the technical methods used are given in the supporting information (text S1).

## Examples Using the Basic Analysis

We have applied these methods to four examples of prior distributions for the intrinsic conception rate. For examples 1 to 3, as in [31] it is assumed that a small proportion of the population is sterile, i.e. has zero probability of conceiving spontaneously, with the remainder of couples having intrinsic conception rates drawn from a beta distribution. This is a continuous distribution, with a range of 0 to 1. Varying the two parameters that define the distribution changes the mean and variance, and, as in this study, the distribution is widely used as a prior distribution for modelling proportions [32]. A formula for the beta distribution is given in the supporting information (text S1). Examples 1 and 3 were chosen to give approximate fits to the results respectively reported by Gnoth et al. [1] and Wang et al. [2], with achievement of a clinical pregnancy as the relevant end-point. Example 2 was chosen to illustrate the effect of modifying the distribution of example 1 to make the population systematically less fertile. Example 4 illustrates a hypothetical population in which those couples which are not sterile have a distribution of intrinsic conception rates described by a mixture of two beta distributions, to represent a population containing a high-fertility subpopulation and a low-fertility subpopulation. The main purpose of the four examples is to illustrate how fertility metrics, potentially relevant to assessment and treatment decisions, can be calculated as a function of the number of cycles of non-conception using the methods described below.

**Figure 2:**
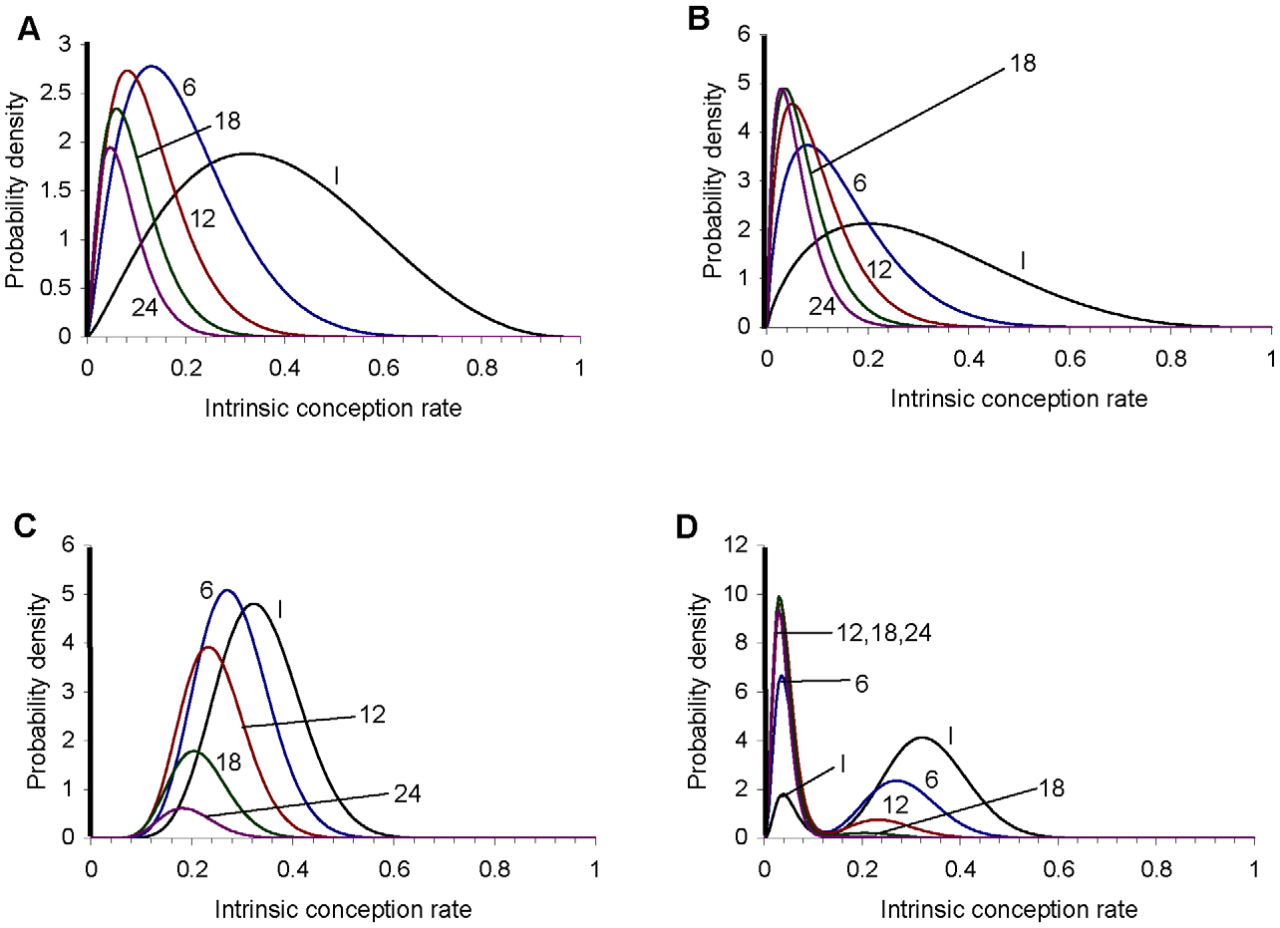
Distribution of the intrinsic conception rate for examples 1 to 4: (A) example 1, (B) example 2, (C) example 3, (D) example 4. In each panel the plot labelled I shows the initial (prior) distribution. The other plots show the distribution conditional on non-conception after 6, 12, 18 and 24 cycles. A thick line represents the finite proportion of the population with an intrinsic conception rate of zero. (This is intended as a schematic representation: the thickness of the line is not proportional to the proportion of couples who are sterile.) The total area underneath each curve corresponds to the proportion of the remaining population which is not sterile: this decreases with the number of cycles as the proportion of the remaining population who are sterile increases (see first column of tables 2, 3, 4, and 5). For example 4, the part of the distribution representing the low fertility segment of the population (i.e. with an intrinsic conception rate below about 0.12) changes little as the number of cycles of non-conception increases from 12 to 24, while the proportion of the remaining population in the higher fertility segment (i.e. with an intrinsic conception rate above about 0.12) is very low after 18 cycles and negligible after 24 cycles.

Leridon [24] provides estimates of the prevalence of sterile couples, ranging from 2.3% at 25 years of (female) age to 6.0% at 30 years and 14% at 35 years. We have assumed 5% sterility for examples 1, 2 and 4. For example 3, it was found to be necessary to assume a much lower level of sterility to achieve a good fit to the data given by Wang et al. [2]; we assumed 1% sterility. The full parameters for the four examples are as follows:

Example 1: 5% of couples are assumed to be sterile, therefore having an intrinsic conception rate of zero. The remaining 95% are assumed to have intrinsic conception rates drawn from a beta distribution with parameters α = 2.3 and β = 3.7 (see the supporting information: text S1). The distribution is illustrated in Figure 2(a).

Example 2: 5% of couples are assumed to have a zero probability of conceiving spontaneously. The remaining 95% again are assumed to have intrinsic conception rates described by a beta distribution, but for this example the parameters are α = 1.8 and β = 4.2. Example 2 deliberately describes a less fertile population than that in example 1: whilst 5% of the population are sterile in both examples, for the upper 95% of the distribution all percentiles of the prior distribution of the intrinsic conception rate are lower for population 2 than for population 1; we have verified this numerically from the probability density functions. The distribution is illustrated in Figure 2(b).

Example 3: 1% of couples are assumed to have an intrinsic conception rate of zero. The remaining 99% of the population are assumed to have intrinsic conception rates described by a beta distribution, with α = 11 and β = 22. (Figure 2(c)).

Example 4. 5% of couples are assumed to have an intrinsic conception rate of zero; 85% have intrinsic conception rates drawn from a beta distribution with α = 11 and β = 22 (as used in example 3); and 10% have intrinsic conception rates drawn from a beta distribution with α = 4 and β = 76. This example can be conceptualised as a mixture of distinct fertile and subfertile populations; it has two separate peaks in the distribution of the intrinsic conception rate of the non-sterile population (Figure 2(d)).

### Results

For examples 1, 2, 3, and 4, Figure 2 shows the distributions of the intrinsic conception rate after different numbers of cycles of attempted conception without success. Tables 2, 3, 4 and 5 respectively show, after 0, 1, 3, 6, 9, 12, 18, 24 and 36 elapsed cycles without conception, various metrics which may be relevant to determining whether a couple should receive medical assessment/treatment or else continue trying to conceive without medical intervention. The last column of each table shows the cumulative probability of conception. This is equal to 1−*s*(*n*), where *s*(*n*) is given in equation (3). The supporting information (text S1) gives fuller versions of these tables showing the same metrics, together with the probability of conceiving in the following 24 cycles, for all values of number of cycles elapsed from 0 to 36.

**Table 2:** Fertility metrics as a function of the number of cycles of attempted conception for example 1.

Number of cycles elapsed	Proportion of remaining population who are sterile	Median intrinsic conception rate in remaining population	90^th^ percentile of intrinsic conception rate in remaining population	Probability of conceiving in next cycle	Probability of conceiving in next 12 cycles	Cumulative probability of conception
0	0.050	0.356	0.633	0.364	0.906	0.000
1	0.079	0.292	0.551	0.303	0.861	0.364
3	0.151	0.205	0.431	0.217	0.758	0.670
6	0.282	0.122	0.314	0.138	0.596	0.823
9	0.414	0.062	0.237	0.090	0.452	0.879
12	0.530	0.000	0.183	0.060	0.337	0.906
18	0.699	0.000	0.112	0.029	0.189	0.928
24	0.800	0.000	0.067	0.015	0.111	0.938
35	0.895	0.000	0.012	0.006	0.048	0.944
36	0.900	0.000	0.000	0.005	0.045	0.944

In example 1: 5% of couples are assumed to be sterile (intrinsic conception rate = 0). The remaining 95% are assumed to have intrinsic conception rates described by a beta distribution with parameters α = 2.3 and β = 3.7.

**Table 3:** Fertility metrics as a function of the number of cycles of attempted conception for example 2.

Number of cycles elapsed	Proportion of remaining population who are sterile	Median intrinsic conception rate in remaining population	90^th^ percentile of intrinsic conception rate in remaining population	Probability of conceiving in next cycle	Probability of conceiving in next 12 cycles	Cumulative probability of conception
0	0.050	0.264	0.539	0.285	0.856	0.000
1	0.070	0.218	0.467	0.239	0.812	0.285
3	0.116	0.157	0.365	0.177	0.724	0.567
6	0.192	0.103	0.270	0.121	0.601	0.740
12	0.348	0.046	0.169	0.065	0.407	0.856
18	0.482	0.010	0.116	0.039	0.277	0.896
24	0.587	0.000	0.083	0.025	0.193	0.915
36	0.728	0.000	0.046	0.012	0.101	0.931

In example 2, 5% of couples are assumed to have a zero probability of conceiving. The remaining 95% are assumed to have intrinsic conception rates described by a beta distribution, with α = 1.8 and β = 4.2. This population is systematically less fertile than that in example 1.

**Table 4:** Fertility metrics as a function of the number of cycles of attempted conception for example 3.

Number of cycles elapsed	Proportion of remaining population who are sterile	Median intrinsic conception rate in remaining population	90^th^ percentile of intrinsic conception rate in remaining population	Probability of conceiving in next cycle	Probability of conceiving in next 12 cycles	Cumulative probability of conception
0	0.010	0.329	0.440	0.330	0.973	0.000
1	0.015	0.319	0.427	0.319	0.966	0.330
3	0.032	0.299	0.404	0.296	0.945	0.684
6	0.086	0.270	0.372	0.258	0.884	0.884
9	0.198	0.237	0.341	0.210	0.768	0.950
12	0.375	0.190	0.308	0.153	0.593	0.973
18	0.745	0.000	0.228	0.055	0.236	0.987
24	0.921	0.000	0.000	0.015	0.071	0.989
36	0.992	0.000	0.000	0.001	0.007	0.990

In example 3, 1% of couples are assumed to have an intrinsic conception rate of zero. The remaining 99.5% of the population are assumed to have intrinsic conception rates described by a beta distribution, with α = 11 and β = 22.

**Table 5:** Fertility metrics as a function of the number of cycles of attempted conception for example 4.

Number of cycles elapsed	Proportion of remaining population who are sterile	Median intrinsic conception rate in remaining population	90^th^ percentile of intrinsic conception rate in remaining population	Probability of conceiving in next cycle	Probability of conceiving in next 12 cycles	Cumulative probability of conception
0	0.050	0.312	0.432	0.288	0.879	0.000
1	0.070	0.294	0.416	0.264	0.839	0.288
3	0.125	0.250	0.385	0.212	0.735	0.601
6	0.232	0.062	0.332	0.135	0.553	0.785
9	0.335	0.036	0.270	0.080	0.398	0.851
12	0.415	0.024	0.181	0.049	0.297	0.879
18	0.519	0.000	0.061	0.025	0.198	0.904
24	0.590	0.000	0.051	0.017	0.152	0.915
36	0.696	0.000	0.040	0.011	0.101	0.928

In example 4, 5% of couples are assumed to have an intrinsic conception rate of zero; 85% have intrinsic conception rates drawn from a beta distribution with α = 11 and β = 22; and 10% have intrinsic conception rates drawn from a beta distribution with α = 4 and β = 76.

**Figure 3:**
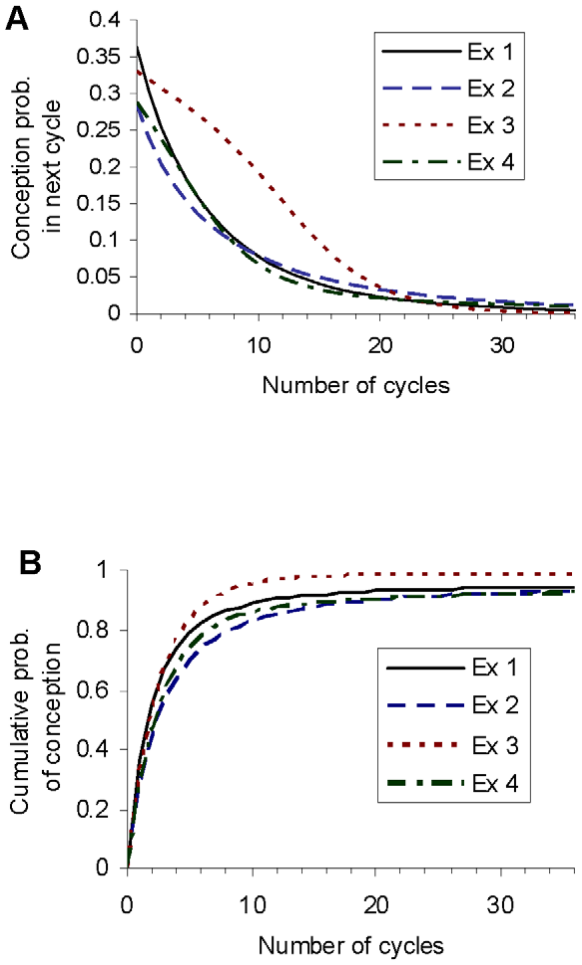
For examples 1 to 4 this shows: (A) the probability of conceiving on the next cycle for a couple who have not yet conceived, and (B) the cumulative conception probability. They are plotted as a function of the number of cycles of attempted conception.

The results for example 1, shown in Table 2, are in good agreement with cumulative probabilities of conception (achievement of a clinical pregnancy) reported by Gnoth et al. [1] (Table I, for all couples). The results for example 3, shown in Table 4 are in good agreement with conception rates reported by Wang et al. [2], Table 2; for consistency in the analysis we have again considered conception to be a clinical pregnancy). Example 2 was devised primarily to illustrate the effect of making the population in example 1 systematically less fertile, but the parameters of the distribution were also chosen to give an approximate fit to the cumulative conception probabilities quoted by the UK's National Institute for Health and Clinical Excellence of 84%, 92% and 93% after 1, 2 and 3 years respectively [33]; the results shown in Table 3 are in reasonable agreement with these cumulative conception probabilities. Figure 3(a) shows how the probability of conception on the next cycle depends on the number of elapsed cycles without conception, for the four examples. In all four examples the probability of conception in the next cycle declines with the number of elapsed cycles, but the pattern of decline varies among the examples. Comparing examples 1 and 2, the probability of conceiving on the first cycle (i.e. for number of elapsed cycles = 0) is higher for example 1: this is a straightforward consequence of the mean intrinsic conception rate in the prior population being higher for example 1 than for example 2. However, the probability of conceiving on the next cycle declines more rapidly with the number of cycles elapsed for example 1 than for example 2, so that the two curves cross. This is because example 1 has more high fertility couples, who will tend to achieve a conception and therefore leave the population relatively soon, whereas example 2 has more low to intermediate fertility couples who will tend to stay in the population longer. Hence, the proportion of couples who are sterile is initially 5% for both examples, but this increases as a proportion of the remaining population more rapidly for example 1 than for example 2 (compare Table 2 and Table 3) because more couples in example 1 are conceiving and leaving the population. Example 3 shows a somewhat different pattern: the initial proportion of couples who are sterile is only 1%, and the remaining (fertile) population has a low variance of fertility, with almost all couples having an intrinsic conception rate below 0.6. The probability of conception on the next cycle therefore stays at a reasonably high level as long as the proportion of the remaining population who are sterile remains small. However, the relative lack of couples with low to intermediate intrinsic conception rates in this population means that the fertile members tend to become pregnant and leave the population relatively quickly, so that the proportion of the population who are sterile goes through a relatively sudden increase, leading to a relatively abrupt and rapid decline in the probability of conception on the next cycle, this probability eventually falling below that for the other examples. Example 4 gives a probability of conception on the first cycle which is very similar to that for example 2, reflecting similar mean intrinsic conception rates for the prior populations, but over the first few cycles the probability of conception declines more rapidly for example 2, because the variance in the prior population's intrinsic conception rate in greater for example 2 than for example 4. However, the probability of conception in the next cycle for example 4 eventually falls below that for example 2; this is because In example 4 there is a relatively homogeneous high fertility subpopulation which leaves the population (by achieving a pregnancy) relatively quickly, leaving mostly very low fertility couples behind, whereas example 2 has more low to medium fertility couples; these tend to remain in the population for a larger number of cycles than high-fertility couples. Cumulative conception probabilities for the four examples are shown in Figure 3(b). There is a clear ordering over most of the range of number of elapsed months, with cumulative conception probabilities being generally highest for example 3, reflecting both the low sterile population and the highly fertile non-sterile population, then example 1, then example 2, and lastly example 4.

Table 6 shows the number of cycles of non-conception which must elapse before different possible metrics of subfertility are reached for the four examples. All the metrics are reached last in example 3. This is because, of the four examples, example 3 has the lowest proportion of sterile couples in the prior population (1%, compared to 5% for the others) and has the most homogeneous non-sterile population. The homogeneity of the non-sterile population means that a given metric of subfertility is reached only when the sterile proportion of the remaining population of couples becomes relatively large. Because the initial proportion of sterile couples is small, this transition happens late. All but one of the metrics are reached first for example 4, reflecting the significant low-fertility subpopulation in that example. With example 2 representing a systematically less fertile population than example 1, it may seem surprising that some metrics are reached sooner for example 1 than for example 2. The reason is that the higher-fertility couples of example 1 tend to achieve a pregnancy and therefore leave the population relatively quickly, whereas the lower-fertility (but still fertile) couples of example 2 remain in the population longer.

**Table 6:** Number of cycles of attempted conception required for various indicators of subfertility to be attained, for examples 1, 2, 3 and 4.

	Median intrinsic conception rate <0.05	90^th^ percentile of intrinsic conception rate <0.2	Probability of conceiving in next cycle <0.1	Probability of conceiving in next cycle <0.05	Probability of conceiving in next 12 cycles <0.5
Example 1	10	11	9	14	8
Example 2	12	10	8	15	9
Example 3	14	20	15	19	14
Example 4	7	12	8	12	7

The characteristics of the populations for examples 1, 2, 3 and 4 are described in the legends of Tables 2, 3, 4 and 5, and in the text.

It is interesting that, although the number of cycles taken to reach a particular threshold varies with the different examples, the results do not differ greatly. In particular, for each example it is approximately a year before the chance of conceiving in the next cycle falls below 10%.

## Time to Pregnancy with Reproductive Ageing

As couples grow older, their fertility declines. In particular, increasing female age is associated with a decreasing intrinsic conception rate. Female age, therefore, may be expected to have a bearing on how many cycles of attempted natural conception need to elapse before medical investigation and treatment is appropriate. A particular fertility metric could be applied, such as the chance of conceiving in the next cycle falling to below 10%, or the chance of conceiving in the next 12 cycles falling below 50%. When a specific metric is reached, medical intervention may be appropriate. How does the number of non-conception cycles required to reach the threshold metric depend upon the couple's female age? To investigate this question, we considered a population fertility model which explicitly includes the effects of declining fertility with female age. The model uses data presented by Leridon [24].

Example 5: For this example, it is assumed that couples vary in the female age at which they become sterile. As female age increases, the proportion of couples who are sterile increases. We use the sterility data given in Leridon [24], Table 2. Specifically: 1% of couples are already sterile at a female age of 25; the median female age at which sterility occurs is between 44 and 45; and at a female age of 59 all couples are sterile. A couple's fertility is assumed to decline linearly for 12.5 years prior to sterility [24]; before this decline, it is at its peak value. (For example, a couple which becomes sterile at a female age of 45 will be at peak fertility up to a female age of 32.5, and then have a steadily declining intrinsic conception rate until sterility occurs.) Peak fertility varies among couples according to a Beta distribution with parameters α = 3 and β = 10 [34], giving a mean peak intrinsic conception rate of 0.23.

We have incorporated this model of declining fertility into the computational analysis. Details of the technical methods used are given in the supporting information (text S1).

### Results

**Figure 4:**
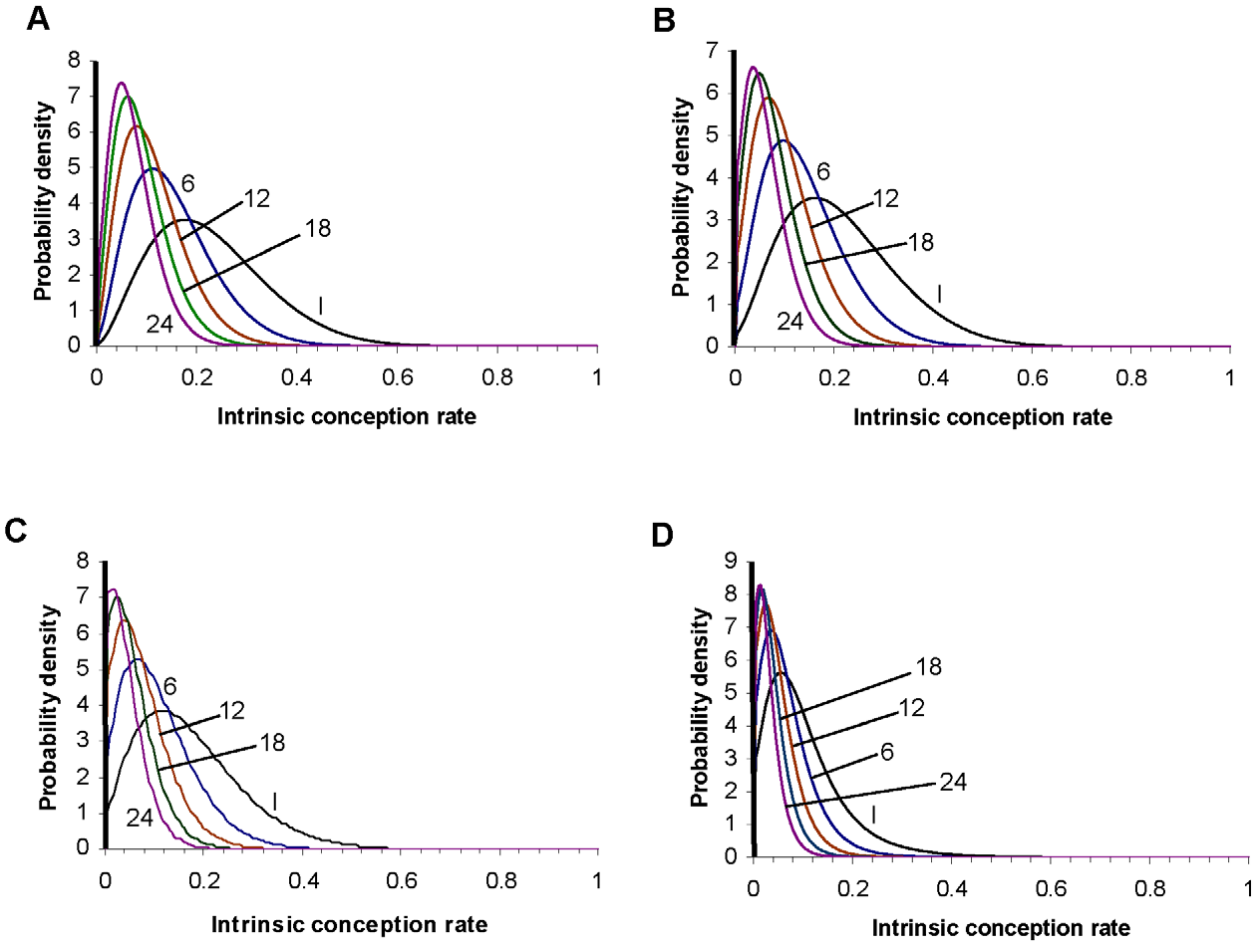
Distribution of the intrinsic conception rate at 4 different female ages at the start of the attempt to conceive, for example 5: (A) age 25, (B) age 30, (C) age 35, (D) age 40. In each panel the plot labelled I shows the initial (prior) distribution. The other plots show the distribution conditional on non-conception after 6, 12, 18 and 24 cycles. A thick line represents the finite proportion of the population with an intrinsic conception rate of zero. The total area underneath each curve corresponds to the proportion of the remaining population which is not sterile.

**Figure 5:**
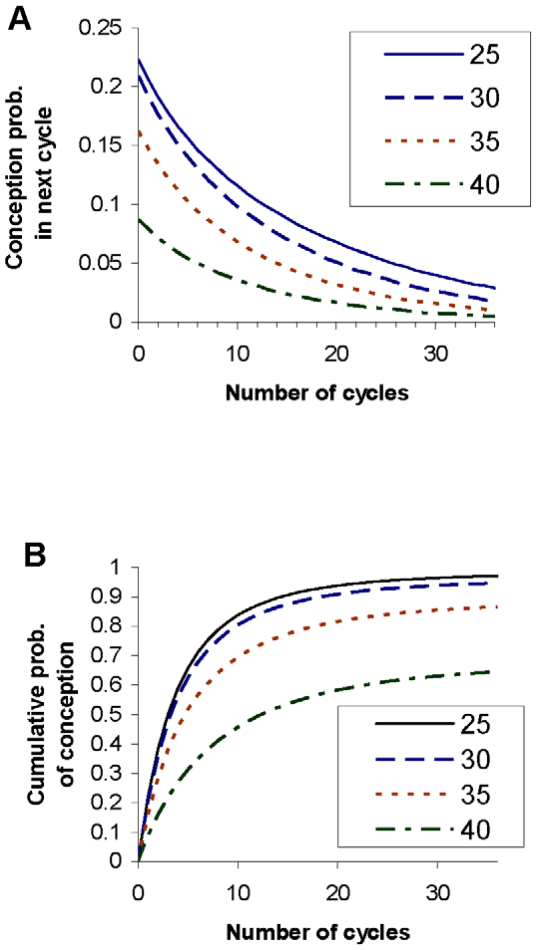
For example 5 this shows, for female ages at the start of the attempt to conceive, of 25, 30, 35 and 40: (A) the probability of conceiving on the next cycle for a couple who have not yet conceived, and (B) the cumulative conception probability. They are plotted as a function of the number of cycles of attempted conception.

We have run the computational analysis of time to pregnancy for example 5, for female ages of 25, 30, 35 and 40. Figure 4 shows the distributions of the intrinsic conception rate after different numbers of cycles of non-conception, for the different ages. Figure 5 shows the probability of conception on the next cycle as a function of the number of cycles that have elapsed without conception, and the cumulative conception probability, for different female ages at which a couple start trying to conceive. Tables 7, 8, 9, and 10 show fertility metrics after different numbers of elapsed cycles, for couples beginning the attempt to conceive at female ages of 25, 30, 35 and 40 respectively. The supporting information (text S1) gives fuller versions of these tables showing the same metrics, together with the probability of conceiving in the following 24 cycles, for all values of number of cycles elapsed from 0 to 36.

**Table 7:** Fertility metrics as a function of the number of cycles of attempted conception for example 5, at a female age of 25.

Number of cycles elapsed	Proportion of remaining population who are sterile	Median intrinsic conception rate in remaining population	90^th^ percentile of intrinsic conception rate in remaining population	Probability of conceiving in next cycle	Probability of conceiving in next 12 cycles	Cumulative probability of conception
0	0.01	0.21	0.38	0.223	0.872	0
1	0.013	0.193	0.354	0.206	0.853	0.223
3	0.021	0.166	0.311	0.178	0.812	0.501
6	0.036	0.136	0.262	0.146	0.75	0.712
9	0.058	0.113	0.225	0.122	0.687	0.815
12	0.086	0.096	0.196	0.103	0.626	0.872
18	0.159	0.069	0.153	0.075	0.507	0.928
24	0.249	0.048	0.123	0.055	0.4	0.952
36	0.467	0.009	0.08	0.029	0.235	0.971

In example 5, couples are assumed to have peak intrinsic conception rates drawn from a beta distribution with α = 3 and β = 10; the distribution of female ages at which couples become sterile is as given in [24], Table 2; a couple's intrinsic conception rate declines linearly for 12.5 years prior to sterility.

**Table 8:** Fertility metrics as a function of the number of cycles of attempted conception for example 5, at a female age of 30.

Number of cycles elapsed	Proportion of remaining population who are sterile	Median intrinsic conception rate in remaining population	90^th^ percentile of intrinsic conception rate in remaining population	Probability of conceiving in next cycle	Probability of conceiving in next 12 cycles	Cumulative probability of conception
0	0.02	0.195	0.367	0.209	0.839	0
1	0.026	0.178	0.341	0.191	0.814	0.209
3	0.04	0.151	0.297	0.163	0.763	0.473
6	0.068	0.119	0.246	0.13	0.685	0.677
9	0.105	0.096	0.208	0.105	0.607	0.781
12	0.149	0.077	0.179	0.086	0.532	0.839
18	0.259	0.048	0.134	0.058	0.398	0.898
24	0.379	0.024	0.102	0.039	0.29	0.925
36	0.606	0	0.059	0.018	0.148	0.947

In example 5, couples are assumed to have peak intrinsic conception rates drawn from a beta distribution with α = 3 and β = 10; the distribution of female ages at which couples become sterile is as given in [24], Table 2; a couple's intrinsic conception rate declines linearly for 12.5 years prior to sterility.

**Table 9:** Fertility metrics as a function of the number of cycles of attempted conception for example 5, at a female age of 35.

Number of cycles elapsed	Proportion of remaining population who are sterile	Median intrinsic conception rate in remaining population	90^th^ percentile of intrinsic conception rate in remaining population	Probability of conceiving in next cycle	Probability of conceiving in next 12 cycles	Cumulative probability of conception
0	0.051	0.147	0.31	0.162	0.733	0
1	0.062	0.133	0.286	0.147	0.7	0.162
3	0.088	0.109	0.245	0.122	0.635	0.381
6	0.133	0.082	0.2	0.094	0.544	0.566
9	0.184	0.062	0.166	0.074	0.46	0.67
12	0.239	0.046	0.139	0.058	0.387	0.733
18	0.365	0.021	0.1	0.037	0.268	0.802
24	0.486	0.002	0.072	0.024	0.184	0.836
36	0.7	0	0.036	0.01	0.085	0.866

In example 5, couples are assumed to have peak intrinsic conception rates drawn from a beta distribution with α = 3 and β = 10; the distribution of female ages at which couples become sterile is as given in [24], Table 2; a couple's intrinsic conception rate declines linearly for 12.5 years prior to sterility.

**Table 10:** Fertility metrics as a function of the number of cycles of attempted conception for example 5, at a female age of 40.

Number of cycles elapsed	Proportion of remaining population who are sterile	Median intrinsic conception rate in remaining population	90^th^ percentile of intrinsic conception rate in remaining population	Probability of conceiving in next cycle	Probability of conceiving in next 12 cycles	Cumulative probability of conception
0	0.166	0.07	0.194	0.087	0.494	0
1	0.185	0.063	0.177	0.079	0.463	0.087
3	0.225	0.051	0.151	0.065	0.406	0.219
6	0.284	0.037	0.123	0.05	0.333	0.35
9	0.344	0.025	0.101	0.039	0.273	0.435
12	0.402	0.015	0.085	0.031	0.223	0.494
18	0.516	0	0.06	0.019	0.147	0.567
24	0.614	0	0.042	0.012	0.096	0.607
36	0.772	0	0.017	0.005	0.038	0.644

In example 5, couples are assumed to have peak intrinsic conception rates drawn from a beta distribution with α = 3 and β = 10; the distribution of female ages at which couples become sterile is as given in [24], Table 2; a couple's intrinsic conception rate declines linearly for 12.5 years prior to sterility.

Figures 4 and 5 show how fertility declines with age in the model. The curves differ little between a female age of 25 and 30, but fertility drops markedly by the time female age reaches 35. By the time female age has reached 40, the probability of conception on the first cycle has fallen to below 0.1. It declines for subsequent cycles, with a cumulative probability of conception within 3 years of approximately 0.64.

Table 11 shows the number of non-conception cycles which elapse before different possible metrics of subfertility are reached, for ages 25, 30, 35 and 40. There is a clear pattern to the results: as female age increases, the number of cycles for all subfertility metrics to be reached decreases. At a female age of 25 or 30, the overwhelming majority of couples are of high fertility, and only a very small proportion are sterile. This means that failure to conceive on the first few cycles is most commonly simply bad luck, and a large number of non-conception cycles need to elapse before the probability that the couple is sterile or subfertile is high enough for a subfertility metric to be reached. At a female age of 35, sterility and subfertility are less rare, so that fewer non-conception cycles need to elapse before a subfertility metric is reached. For example, Table 11 shows that the number of non-conception cycles before the probability of conception in the next cycle falls to below 10% is 13 at age 25, falling to 10 at age 30 and 6 at age 35. At a female age of 40, the probability is below 10% even before the first cycle of attempted conception. Indeed, at a female age of 40, the fertility of a typical couple has declined so far that 3 of our 5 selected metrics of subfertility will have already been reached at the beginning of the attempt to conceive.

**Table 11:** Number of cycles of attempted conception required at different ages for various indicators of subfertility to be attained, for example 5.

	Median intrinsic conception rate <0.05	90^th^ percentile of intrinsic conception rate <0.2	Probability of conceiving in next cycle <0.1	Probability of conceiving in next cycle <0.05	Probability of conceiving in next 12 cycles <0.5
Age 25	24	12	13	26	19
Age 30	18	10	10	21	14
Age 35	12	6	6	15	8
Age 40	4	0	0	6	0

The model of how fertility declines with age for example 5 is described in the text, with details of the computational implementation given in the supporting information (text S1).

For the data used in the model, sterility was defined as an inability to conceive and become pregnant (i.e. to achieve a clinical pregnancy) [24]. Pregnancies that resulted in early loss or miscarriage were included as conceptions. The proportion of conceptions that miscarry increases substantially in women in their late thirties and especially after 40 years of age. Thus the metrics derived from the model may be considered optimistic, since they calculate for occurrence of conception, while live birth will be less likely. For a similar reason, in these data sterility occurs at a relatively high age.

We now consider an important technical question: what effect does reproductive ageing during the time that a couple is trying to conceive have on the calculated number of cycles of attempted conception before various metrics of subfertility are reached? For example 5, we have investigated this by running a modified version of the program, in which there is no reproductive ageing during the period of attempted conception. Table 12 shows the change, resulting from holding reproductive ageing static during the period of attempted conception, in the calculated number of non-conception cycles before each of the five metrics we have considered is reached. It can be seen that in some cases the calculated number of cycles did not change, while in other cases it increased by either one or two cycles. That is, neglecting reproductive ageing during the period of attempted conception will tend to have the effect of overestimating the number of cycles of attempted conception before required subfertility metrics are reached, but not by a large amount.

**Table 12:** Change in the computed number of cycles of attempted conception required for various indicators of subfertility to be attained, as a result of reproductive ageing during the conception process being switched off, for example 5.

	Median intrinsic conception rate <0.05	90^th^ percentile of intrinsic conception rate <0.2	Probability of conceiving in next cycle <0.1	Probability of conceiving in next cycle <0.05	Probability of conceiving in next 12 cycles <0.5
Age 25	+2	0	+1	+2	+1
Age 30	+2	+1	+1	+2	+2
Age 35	+2	+1	0	+2	+2
Age 40	+1	0	0	+2	+1

The model of how fertility declines with age for example 5 is described in the text, with details of the computational implementation given in the supporting information (text S1).

In using this model of reproductive ageing, it should be recognised that exactly how fertility declines for an individual couple is not known. To model a population requires assumptions about (a) the changing fertility profile over time for an individual couple, and (b) the variation among couples in the parameters describing this profile. Leridon's model [24], on which our analysis is based, gives a complete description of (a) and (b); to our knowledge it is the most complete model of reproductive ageing published to date. It replicates empirical results about ageing at a population level. It does not necessarily follow, however, that the model accurately captures how fertility declines for an individual couple. Different combinations of assumptions for (a) and (b) may yield the same outcome at a population level.

However, we believe that the general pattern of the results for example 5 is likely to hold for any reasonable assumptions about reproductive ageing in a human population.

## Discussion

Couples vary in their fertility. A model of conception has been used to shed light on the following question: what can be deduced about a couple's fertility from the duration of their attempt to conceive? When a couple starts trying for a baby, there will be some uncertainty in their intrinsic conception rate, i.e. their probability of conception per cycle. In our modelling framework, this uncertainty is described by a prior probability distribution for the intrinsic conception rate; this distribution will depend on the population from which the couple is drawn. We have developed a numerical computational method for analysing attempted conception, for any chosen prior distribution of the intrinsic conception rate, over any specified number of cycles. For couples who do not conceive over these cycles, the program calculates a posterior distribution of the intrinsic conception rate, together with various fertility metrics, potentially relevant to clinical decision-making.

For the basic analysis, it is assumed that each couple's intrinsic conception rate can, for modelling purposes, be treated as constant over the period of time being considered. Results have been computed for four examples of prior distributions of the intrinsic conception rate. For each example it has been assumed that a small proportion of couples are sterile, and the remainder vary continuously in their intrinsic conception rate. Examples 1 to 3 produce conception patterns over time which are consistent with those given in [1], [33] and [2] respectively. Our findings give a plausible indication of how differences between the conception patterns of different populations may be understood as consequences of differences in distributions of the intrinsic conception rate. These differences between populations indicate that it is important not to over-generalise findings derived from one particular population. For example, Gnoth et al. [1] considered a relatively fertile population: couples had a mean female age of 29.0 and subjects were practising fertility-awareness methods, while the study population excluded some couples with previous fertility problems. Furthermore, the study population is likely to have contained more couples of relatively high socio-economic position than the general population (C. Gnoth, personal communication). Wang et al. [2] considered textile workers, so subjects were not of high socio-economic status, but the mean female age of 24.9 would tend to imply high fertility, while the fact that these relatively young women were all married suggests a likelihood of a lower level of sexually transmitted diseases, and potentially a low level of fertility problems associated with their consequences. Caution is needed in making a direct comparison between East Asian and Western populations; there is, for example, evidence to suggest that there are regional differences in conception rates among European populations [35]. Nevertheless, it is not entirely surprising that the cumulative conception rates stated by the UK's National Institute for Health and Clinical Excellence [33] are lower than those reported by both Gnoth et al. [1] and Wang et al. [2].

When a clinician is faced with a couple, how will he or she know to which population the couple belongs? The answer is probabilistic: if the couple belong to one of several populations, but it is not known which, then they can be treated as a random draw from a composite population, given by the appropriately weighted mixture of the component populations. Example 4 was devised to illustrate such a composite population.

For clinical decision-making, the important question is: how many cycles of non-conception should be taken as an indicator that a couple needs investigation and treatment? As this analysis has shown, it is necessary to consider *probabilistic* measures of subfertility. In Table 6 we have considered five such measures for each of these four examples. The table shows that the number of cycles required for a subfertility metric to be attained depends on both the metric chosen and the prior distribution of the intrinsic conception rate. It also suggests, however, that the notion of 12 cycles of non-conception as an indicator of subfertility is moderately robust: none of the measures on any of the examples yields a number of cycles that differs greatly from this number.

For these examples we did not use a formal mathematical optimisation process to fit distributions to data. Bongaarts [18] used a search procedure to find a best fit beta distribution. However, more than one general form of distribution may give a conception pattern that is consistent with a given dataset. In the supporting information (text S1) additional examples are presented in which the non-sterile population is described by a continuous triangular distribution (see for example Potter [36]) or a compressed beta distribution with a maximum intrinsic conception rate well below 1. The parameters of these additional examples were chosen to give a reasonable fit to the same data to which examples 1 to 3 were fitted. The resulting numbers of cycles for a given metric of subfertility to be reached do not change greatly. Further discussion arising from these additional examples is given in the supporting information.

Female age is of particular importance to a couple's conception prospects [14], [15]. To investigate this in more detail, we modified our computational analysis to incorporate a model of fertility declining with female age (Example 5), including fertility decline during the time period of attempted conception. As expected, the results show that a couple's conception prospects decline with increasing female age. The number of non-conception cycles, before a specified metric of subfertility is reached, also decreases with increasing female age. This is true of all five of the metrics we examined. These results tend to indicate that a couple in which the woman is in her late thirties merits prompter intervention than one in which the woman is in her twenties. Of course, decision making should not depend only on probabilistic measures of a couple's chance of conceiving without medical assistance. It requires, in principle, a full analysis of the costs and benefits of medical treatment, as a function of when treatment is carried out, taking account of the couple's medical history.

These results for female ageing raise the question of whether reproductive ageing during the time period of attempted conception should be incorporated more generally into computational modelling of time to pregnancy in different populations. It should be noted first that the computations required to incorporate reproductive decline after each cycle of non-conception in example 5 required orders of magnitude more computational time than examples 1 to 4. A more fundamental difficulty is that the model of ageing used in example 5 is based on a specific population model [24], with assumptions about both individual ageing and variation within the population; the question of exactly how to tailor the model to fit other populations then arises. There is also the question of how accurately the population-based model captures individual female reproductive ageing. However, the results we report in Table 12 indicate that, at least for the specific assumptions of model 5, the effect of ignoring reproductive decline during the period of attempted conception is modest, adding up to two cycles to the computed number of non-conception cycles before a subfertility metric is reached. It is likely that the magnitude of this effect will be similar for other models making any reasonable assumptions about reproductive ageing in individuals and within populations. This raises the possibility of introducing a simple correction for ageing into any clinical application of the methods described here.

Further empirical and theoretical work on reproductive ageing would be useful. This may draw on IVF studies [37] together with models and measurements of relevant aspects of the female reproductive system [26], [27].

A number of variables other than age are also associated with conception probability, such as previous fertility history of each member of the couple [38]; where available, such data may further reduce uncertainty about a couple's fertility. Where there is a medical condition that influences the intrinsic conception rate (e.g. type 1 or type 2 diabetes [39]), this can be taken into account by estimating a distribution for the intrinsic conception rate for people with the condition. It would then be possible to specify how many cycles of non-conception should precede medical intervention for couples in which the condition is present. Such personalised assessment is increasingly sought, with a view to reducing uncertainty in predicting the outcome of treatments, and allowing the optimisation of healthcare. This is likely to be a particularly useful area for application of the methods described here. The Bayesian methodology means that probabilistic assumptions about how the presence of a disorder influences a person's fertility are made explicit [40].

The modelling approach, as applied to all five examples, assumes that successive cycles of attempted conception are independent. That is, it is assumed that there is no correlation in successive cycles between temporary random factors that may affect the couple's probability of conceiving. Because of this assumption, the approach described here cannot in its present formulation properly represent a situation in which one member of a couple has a disorder which causes temporary subfertility and may last for several months, but has a non-trivial probability of spontaneously dissipating so that fertility is restored, such as, for example, low-weight-related anovulation. This could be captured by incorporating into the model a dynamic process representing how a couple's fertility status evolves over time, e.g. as a Markov process [17], [18].

Simulation can be a useful tool for evaluating outcomes of models of biological processes, including attempted conception over time [34]. The computational tools we have developed in this study, instead use numerical methods to track a population over successive cycles of attempted conception. The main strength of our approach is that posterior probability distributions of the intrinsic conception rate can more easily be computed and plotted, and probability calculations can readily be carried out to a high degree of accuracy.

The methods described in this study may be helpful for the further development of decision support systems in fertility assessment, with potential benefits to patients and clinicians, and to health service funders who must allocate resources through decisions that affect large numbers of patients. We are happy to make the software developed for this study available to readers who wish to explore other examples of prior distributions and to develop these methods further.

Finally we note that, whilst this study has considered the application of uncertainty in intrinsic conception rates to non-medically assisted conception, the finding that the outcomes of repeat IVF cycles for the same patients are positively correlated [37] indicates that these methods are also applicable to tools predicting the probability of live births in couples undergoing intrauterine insemination and in vitro fertilisation [41]. Moreover, this methodology may also be relevant to epidemiology and decision-making in other areas of medicine where there are time-dependent processes with rates which may be heterogeneous within a population. A possible example is the spontaneous clearance of infectious disease [42]–[45]: the methods presented in this study may facilitate, for example, estimation of the probability of spontaneous clearance within some period conditional on spontaneous clearance not having occurred within some previous time period.

## Supporting Information

Text S1 (PDF)
