# Supplementary material for: Time to Pregnancy: A Computational Method for Using the Duration of Non-Conception for Predicting Conception
Source: PLoS One. 2012 Oct 4;7(10):e46544. doi: 10.1371/journal.pone.0046544 (PMC3464305; doi:10.1371/journal.pone.0046544)
Supplement: Text S1 — (PDF) [file pone.0046544.s001.pdf]

Plos ONE: Supporting Information

Time to pregnancy: a computational method for using the duration of non-conception for predicting conception

Peter D Sozou and Geraldine M Hartshorne

Text S1

Appendix A defines the beta distribution, used in examples 1, 2, 3 and 4 in the main text and in the additional examples S1.4, S1.5 and S1.6 presented below in Appendix B.

Appendix B presents and analyses six additional examples of prior distributions of the intrinsic conception rate, and includes discussion arising from these additional examples.

Appendix C describes the mathematical and computational methods used in this study.

Appendix D presents fuller versions of tables giving fertility metrics for all the examples considered. These are: Tables 2 to 5 and 7 to 10 in the main text, for examples 1 to 5; and tables S1.1 to S1.6 in Appendix B, for the additional examples S1.1 to S1.6.

Appendix A: the beta distribution and compressed beta distribution

The beta distribution has two parameters, α and β. The probability density function is given by

$$f(x) = \frac{x^{\alpha-1}(1-x)^{\beta-1}}{\mathrm{B}(\alpha,\beta)}$$

where $\mathrm{B}(\alpha,\beta)$ is the beta function and serves as a normalisation constant. It is given by

$$\mathrm{B}(\alpha,\beta) = \int_0^1 u^{\alpha}(1-u)^{\beta}\,du$$

Detailed properties of the beta distribution are given in Forbes et *al*. [32] (reference 32 in the main text). Note that Forbes et *al*. refer to the distribution parameters as ν and ω, rather than α and β; we have chosen (α, β) because this appears to be the more common notation.

For examples 1 to 3 in the main text, the distribution of intrinsic conception rates in the non-sterile population is assumed to be a beta distribution. For example 4 in the main text, the distribution of intrinsic conception rates in the non-sterile population is assumed to be a mixture of two beta distributions.

For additional examples S1.4, S1.5 and S1.6, described in Appendix B, the non-sterile population is drawn from a compressed beta distribution. This is defined as follows: the intrinsic conception rate y is given by $y = kz$, where z has a beta distribution and k is a positive constant, with $k < 1$. This means that the maximum possible value of the intrinsic conception rate within the population is k. For additional examples S1.4, S1.5 and S1.6 we take $k = 0.6$.

Appendix B: additional examples and further discussion

The question of how sensitive results are to the assumed form of the prior distribution of intrinsic conception rates can be explored by examining how the results change when other forms of distribution are considered. Examples S1.1 to S1.3 are qualitatively similar to examples 1 to 3 in the main text, except that the non-sterile population is described by a triangular distribution (see for example Potter [36] (reference 36 in the main text)) rather than a beta distribution. Examples S1.4 to S1.6 are again qualitatively similar to examples 1 to 3 in the main text, except that the non-sterile population is now described by a compressed beta distribution in which the maximum possible intrinsic conception rate is 0.6. Formally, we can say that the intrinsic conception rate is given by $y = 0.6z$, where the probability density function for z has a beta distribution. A simple rationale for using a compressed beta distribution rather than the non-compressed form used in the examples in the main text is that it may be plausible to assume that for even the most fertile couples there is an upper limit to their probability of conception per cycle which is considerably less than one. A compressed beta distribution would arise if, for a couple to conceive on a given cycle, two independent processes must both have a positive outcome, where the success rate among couples of the first process has a beta distribution, and the second process has fixed probability k of success. For examples S1.4 to S1.6 we assume that $k = 0.6$.

The additional examples: specification/parameters

Example S1.1: 5% of couples are assumed to be sterile, i.e. have a zero probability of conceiving without medical assistance; their intrinsic conception rate is zero. The remaining 95% are assumed to have intrinsic conception rates described by a triangular distribution, with intrinsic conception rates ranging from 0 to 0.8 and peak probability density at an intrinsic conception rate of 0.4. Figure S1.1(a) shows the probability density function for the intrinsic conception rate for the initial population, together with the posterior distributions conditional on non-conception after 6, 12, 18 and 24 cycles.

Example S1.2: 5% of couples are assumed to have a zero probability of conceiving without medical assistance. The remaining 95% again are assumed to have intrinsic conception rates described by a triangular distribution, but with intrinsic conception rates now ranging from 0 to 0.5 and peak probability density at an intrinsic conception rate of 0.25. Just as example 2 in the main text described a less fertile population than that in example 1 in the main text, so example S1.2 describes a less fertile population than example S1.1: whilst 5% of the population are identically sterile in the two examples, for the upper 95% of the distribution all percentiles of the prior distribution of the intrinsic conception rate are lower for population represented by example S1.2 than for population represented by example S1.1. Figure S1.1(b) shows the probability density function for the intrinsic conception rate for the initial population, together with the posterior distributions conditional on non-conception after 6, 12, 18 and 24 cycles.

Example S1.3: 1% of couples are assumed to have an intrinsic conception rate of zero. The remaining 99% are assumed to have intrinsic conception rates described by a triangular distribution, with intrinsic conception rates ranging from 0.15 to 0.5 and peak probability density at an intrinsic conception rate of 0.325. Figure S1.1(c) shows the probability density function for the intrinsic conception rate for the initial population, together with the posterior distributions conditional on non-conception after 6, 12, 18 and 24 cycles.

Example S1.4: 5% of couples are assumed to have an intrinsic conception rate of zero. The remaining 95% are assumed to have intrinsic conception rates described by a compressed beta distribution, where the intrinsic conception rate is given by $y = 0.6z$, where z has a beta distribution with $\alpha = 1.9$ and $\beta = 1.1$. Figure S1.1(d) shows the probability density function for the intrinsic conception rate for the initial population, together with the posterior distributions conditional on non-conception after 6, 12, 18 and 24 cycles.

Example S1.5. 5% of couples are assumed to have an intrinsic conception rate of zero. The remaining 95% are assumed to have intrinsic conception rates described by a compressed beta

distribution, where the intrinsic conception rate is given by $y = 0.6z$, where z has a beta distribution with $\alpha = 1.4$ and $\beta = 1.6$. Figure S1.1(e) shows the probability density function for the intrinsic conception rate for the initial population, together with the posterior distributions conditional on non-conception after 6, 12, 18 and 24 cycles.

Example S1.6. 1% of couples are assumed to have an intrinsic conception rate of zero. The remaining 99% are assumed to have intrinsic conception rates described by a compressed beta distribution, where the intrinsic conception rate is given by $y = 0.6z$, where z has a beta distribution with $\alpha = 16$ and $\beta = 16$. Figure S1.1(f) shows the probability density function for the intrinsic conception rate for the initial population, together with the posterior distributions conditional on non-conception after 6, 12, 18 and 24 cycles.

Results and discussion for the additional examples

For examples S1.1 to S1.6, figure S1.1 shows the distribution of the intrinsic conception rate conditional on different numbers of cycles of attempted conception without success. Tables S1.1 to S1.6 show, for these examples, fertility metrics after 0, 1, 3, 6, 9, 12, 18, 24 and 36 elapsed cycles without conception. These are the same fertility metrics given in the main text, for examples 1 to 4, in Tables 2 to 5. As in the main text, the last column of each table shows the cumulative probability of conception. Fuller versions of these tables are given in Appendix D: these full tables show the same metrics, together with the probability of conceiving in the following 24 cycles, for all values of number of cycles elapsed from 0 to 36.

The effect of changing the general form of the prior distribution, whilst choosing new parameters to maintain a reasonable fit to the relevant data, can be seen by comparing the relevant figures and tables corresponding to the same data. Specifically, Figures S1.1(a) and (d), and Tables S1.1 and S1.4, should be compared to Figure 2(a) and Table 2 in the original text; Figure S1.1(b) and (e), and Tables S1.2 and S1.5, should be compared to Figure 2(b) and Table 3 in the main text; and Figures S1.1(c) and (f) and Tables S1.3 and S1.6 should be compared to Figure 2(c) and Table 4 in the main text. The general picture from the figures is that, whilst the prior distribution clearly looks different for different forms of prior distribution fitted to the same data, the posterior distributions conditional on six or more cycles of non-conception look more similar.

We now consider more detailed comparisons between examples based on different forms of prior distribution but fitted to the same data.

- Comparison 1, for examples 1, S1.1 and S1.4, fitted to the data given in Gnoth et al. [1] (reference 1 in the main text): Figure S1.2 shows (a) how the probability of conception on the next cycle depends on the number of elapsed cycles, and (b) corresponding cumulative conception probabilities; and Table S1.7 shows how the number of cycles of non-conception which must elapse for the same metrics of infertility or subfertility considered in the main text to be reached (shown in Table 6 for the four examples considered in the main text).
- Comparison 2, for examples 2, S1.2 and S1.5, fitted to the cumulative conception probabilities quoted by the UK's National Institute for Health and Clinical Excellence [33] (reference 25 in the main text) after 1, 2 and 3 years respectively: Figure S1.3 shows (a) how the probability of conception on the next cycle depends on the number of elapsed cycles, and (b) the corresponding cumulative conception probabilities; and Table S1.8 shows how the number of cycles of non-conception which must elapse for metrics of infertility or subfertility to be reached.
- Comparison 3, for examples 2, S1.2 and S1.5, fitted to the conception rates reported by Wang et al. [2] (reference 2 in the main text): Figure S1.4 shows (a) how the probability of conception on the next cycle depends on the number of elapsed cycles, and (b) the corresponding cumulative conception probabilities; and Table S1.9 shows how the number of cycles of non-conception which must elapse for metrics of infertility or subfertility to be reached.

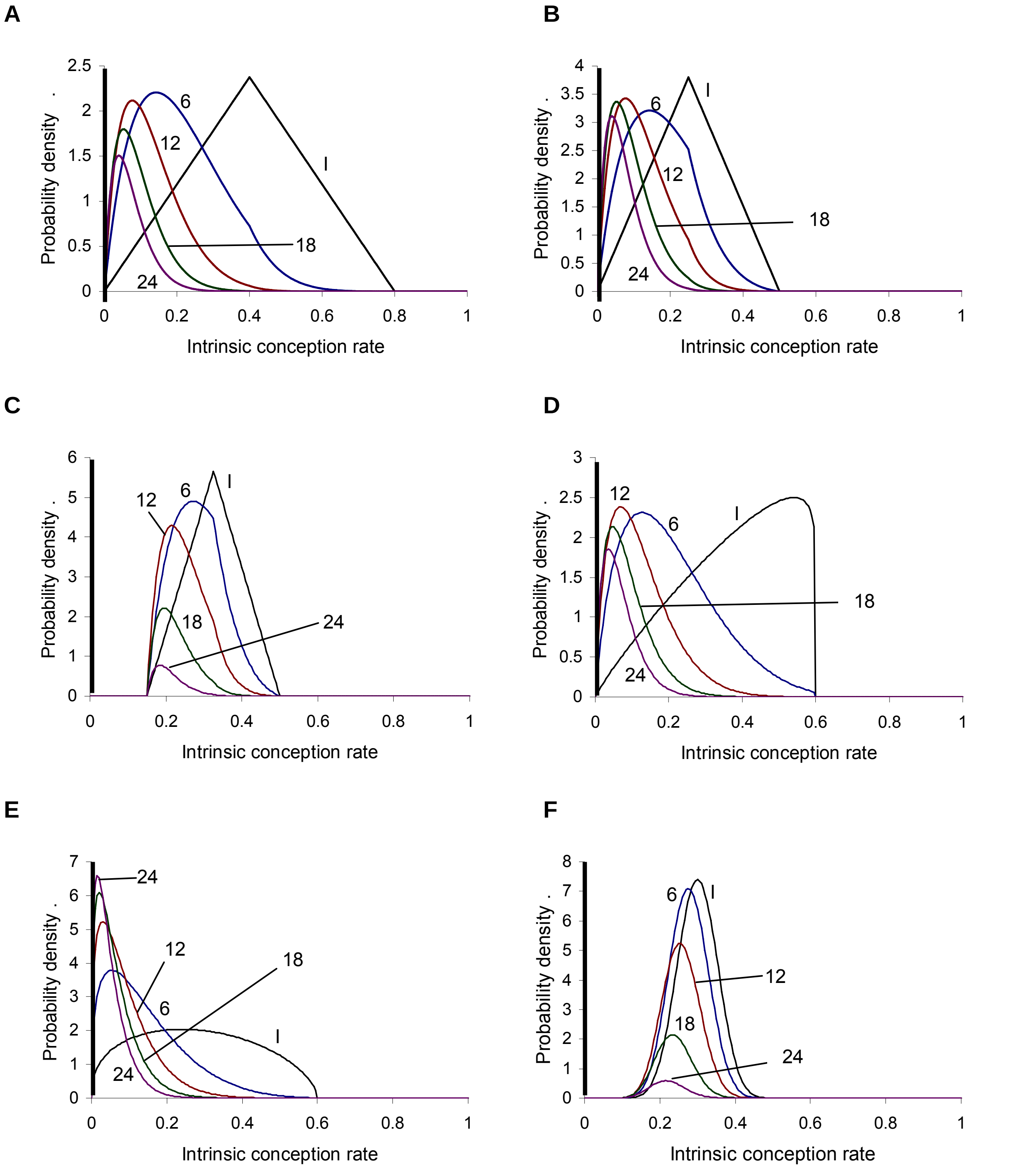

Figure S1.1. Probability distributions for the intrinsic conception rate for: (A) example S1.1, (B) example S1.2, (C) example S1.3, (D) example S1.4, (E) example S1.5, (F) example S1.6. In each case the plot labelled I shows the initial (prior) distribution. The other plots show the distribution conditional on non-conception after 6, 12, 18 and 24 cycles. A thick line represents the finite proportion of the population with an intrinsic conception rate of zero. The total area underneath each curve corresponds to the proportion of the remaining population which is not sterile: this decreases with the number of cycles as those becoming pregnant are lost from the population; consequently the proportion of the remaining population who are sterile increases (see first column of tables S1.1 to S1.6)

Number of cycles elapsed	Proportion of remaining population who are sterile	Median intrinsic conception rate in remaining population	90th percentile of intrinsic conception rate in remaining population	Probability of conceiving in next cycle	Probability of conceiving in next 12 cycles	Cumulative probability of conception
0	0.050	0.389	0.617	0.380	0.917	0.000
1	0.081	0.336	0.559	0.327	0.874	0.380
3	0.166	0.236	0.458	0.237	0.761	0.699
6	0.328	0.120	0.345	0.142	0.570	0.848
9	0.483	0.027	0.249	0.085	0.404	0.896
12	0.606	0.000	0.180	0.052	0.284	0.917
18	0.762	0.000	0.095	0.023	0.147	0.934
24	0.846	0.000	0.048	0.011	0.083	0.941
36	0.922	0.000	0.000	0.004	0.033	0.946

Table S1.1: Fertility metrics as a function of the number of cycles of attempted conception for example S1.1

Number of cycles elapsed	Proportion of remaining population who are sterile	Median intrinsic conception rate in remaining population	90th percentile of intrinsic conception rate in remaining population	Probability of conceiving in next cycle	Probability of conceiving in next 12 cycles	Cumulative probability of conception
0	0.050	0.243	0.385	0.237	0.869	0.000
1	0.066	0.227	0.369	0.221	0.842	0.237
3	0.106	0.192	0.335	0.188	0.778	0.527
6	0.186	0.140	0.286	0.143	0.665	0.732
9	0.283	0.094	0.243	0.106	0.547	0.823
12	0.383	0.056	0.202	0.077	0.438	0.869
18	0.557	0.000	0.134	0.042	0.272	0.910
24	0.682	0.000	0.089	0.024	0.171	0.927
36	0.822	0.000	0.039	0.009	0.076	0.939

Table S1.2: Fertility metrics as a function of the number of cycles of attempted conception for example S1.2

Number of cycles elapsed	Proportion of remaining population who are sterile	Median intrinsic conception rate in remaining population	90th percentile of intrinsic conception rate in remaining population	Probability of conceiving in next cycle	Probability of conceiving in next 12 cycles	Cumulative probability of conception
0	0.010	0.324	0.421	0.322	0.973	0.000
1	0.015	0.316	0.413	0.313	0.967	0.322
3	0.031	0.299	0.396	0.294	0.947	0.675
6	0.083	0.271	0.368	0.259	0.890	0.880
9	0.194	0.239	0.341	0.214	0.777	0.948
12	0.373	0.198	0.311	0.157	0.600	0.973
18	0.756	0.000	0.231	0.056	0.231	0.987
24	0.933	0.000	0.000	0.014	0.063	0.989
36	0.995	0.000	0.000	0.001	0.004	0.990

Table S1.3 Fertility metrics as a function of the number of cycles of attempted conception for example S1.3

Number of cycles elapsed	Proportion of remaining population who are sterile	Median intrinsic conception rate in remaining population	90th percentile of intrinsic conception rate in remaining population	Probability of conceiving in next cycle	Probability of conceiving in next 12 cycles	Cumulative probability of conception
0	0.050	0.388	0.557	0.361	0.913	0.000
1	0.078	0.335	0.538	0.319	0.871	0.361
3	0.159	0.231	0.473	0.236	0.761	0.685
6	0.312	0.116	0.343	0.141	0.572	0.840
9	0.458	0.038	0.243	0.085	0.413	0.891
12	0.576	0.000	0.176	0.053	0.297	0.913
18	0.731	0.000	0.098	0.024	0.161	0.932
24	0.818	0.000	0.054	0.013	0.094	0.939
36	0.903	0.000	0.000	0.005	0.040	0.945

Table S1.4 Fertility metrics as a function of the number of cycles of attempted conception for example S1.4

Number of cycles elapsed	Proportion of remaining population who are sterile	Median fertility in remaining population	90th percentile of fertility in remaining population	Probability of conceiving in next cycle	Probability of conceiving in next 12 cycles	Cumulative probability of conception
0	0.050	0.312	0.432	0.288	0.879	0.000
1	0.070	0.294	0.416	0.264	0.839	0.288
3	0.125	0.250	0.385	0.212	0.735	0.601
6	0.232	0.062	0.332	0.135	0.553	0.785
9	0.335	0.036	0.270	0.080	0.398	0.851
12	0.415	0.024	0.181	0.049	0.297	0.879
18	0.519	0.000	0.061	0.025	0.198	0.904
24	0.590	0.000	0.051	0.017	0.152	0.915
36	0.696	0.000	0.040	0.011	0.101	0.928

Table S1.5: Fertility metrics as a function of the number of cycles of attempted conception for example S1.5

Number of cycles elapsed	Proportion of remaining population who are sterile	Median intrinsic conception rate in remaining population	90th percentile of intrinsic conception rate in remaining population	Probability of conceiving in next cycle	Probability of conceiving in next 12 cycles	Cumulative probability of conception
0	0.010	0.299	0.367	0.297	0.971	0.000
1	0.014	0.295	0.363	0.292	0.965	0.297
3	0.028	0.286	0.355	0.280	0.949	0.645
6	0.073	0.272	0.342	0.257	0.901	0.864
9	0.171	0.252	0.327	0.221	0.802	0.942
12	0.341	0.221	0.308	0.169	0.635	0.971
18	0.741	0.000	0.250	0.061	0.246	0.987
24	0.932	0.000	0.000	0.015	0.063	0.989
36	0.996	0.000	0.000	0.001	0.004	0.990

Table S1.6: Fertility metrics as a function of the number of cycles of attempted conception for example S1.6

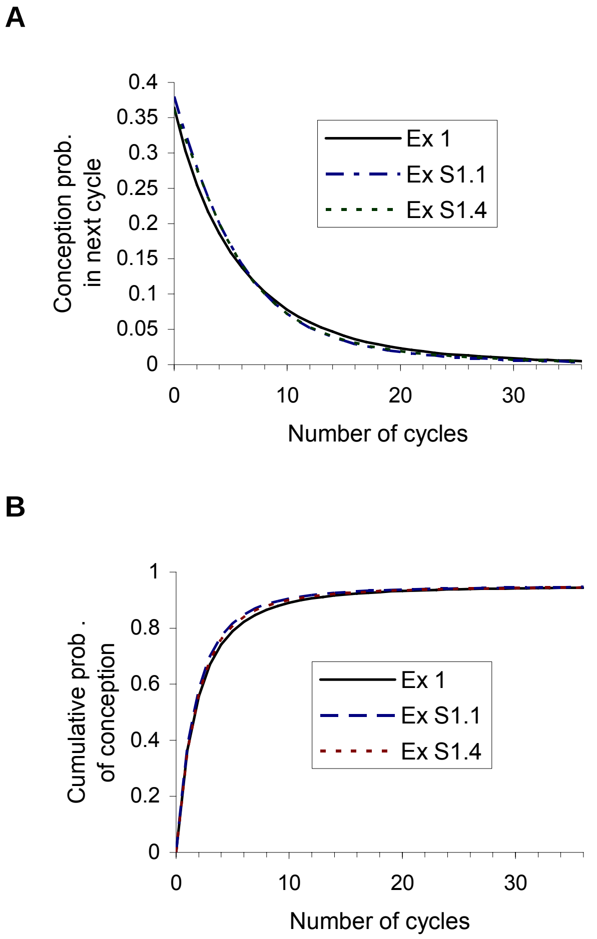

Figure S1.2. This shows, for examples 1, S1.1 and S1.4: (A) probability of conception, conditional on not having already conceived, as a function of the number of elapsed cycles; (B) cumulative conception probability, as a function of number of elapsed cycles.

The general pattern from these comparisons is that different forms of prior distribution fitted to the same dataset tend to give similar patterns of conception over time, i.e. the choice of the parametric form of the prior distribution does not appear to greatly influence the predicted pattern of conception over time. However, there is important variation between these comparisons, with the variation in the patterns of how the probability of conception on the next cycle depends on the number of elapsed cycles being greatest for comparison 2 (contrast Figure S1.3(a) with Figure S1.2(a) and Figure S1.4(a)). Comparison 2 also exhibits variation of up the three cycles in the number of cycles that must elapse for a given metric of subfertility to be reached (Table S1.8), whereas comparisons 1 and 3 exhibit variation of no more than one cycle (Tables S1.7 and S1.9). The difference may be because for comparison 2, comprising examples 2, S1.2 and S1.5, the first data point specifying the proportion of couples who have conceived occurs after 12 cycles. In contrast for comparison 1, comprising examples 1, S1.1 and S1.4, the Gnoth et al. data [1] (Table 1) on which these examples are based gives four data points for the cumulative conception probability up to 12 cycles; and for comparison 3, comprising examples 3, S1.3 and S1.6, the Wang et al. [2] data (Table 2) on which these examples are based gives nine data points for the clinical pregnancy rate up to 12 cycles. Thus, for comparisons 1 and 3 there is more early data which enables the distribution to be "pinned down" more strongly, whereas for comparison 2 the relative lack of early data means that the fitted distribution is less constrained.

A

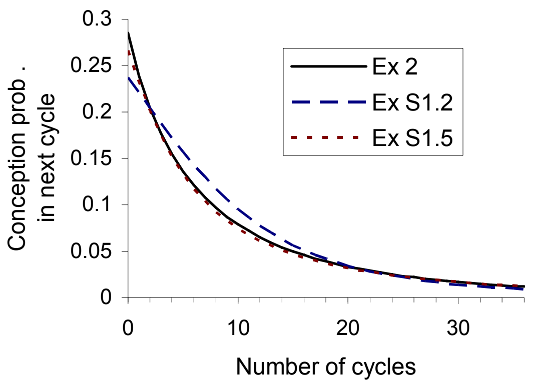

B

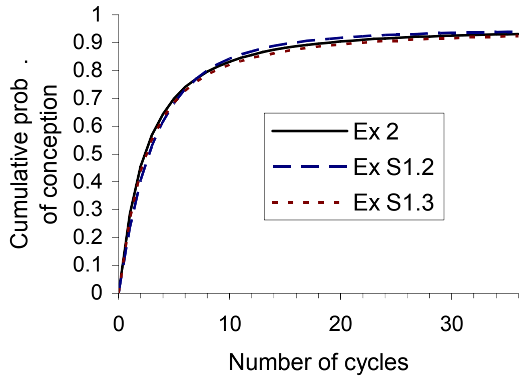

Figure S1.3. This shows, for examples 2, S1.2 and S1.5: (A) probability of conception, conditional on not having already conceived, as a function of the number of elapsed cycles; (B) cumulative conception probability, as a function of number of elapsed cycles.

A

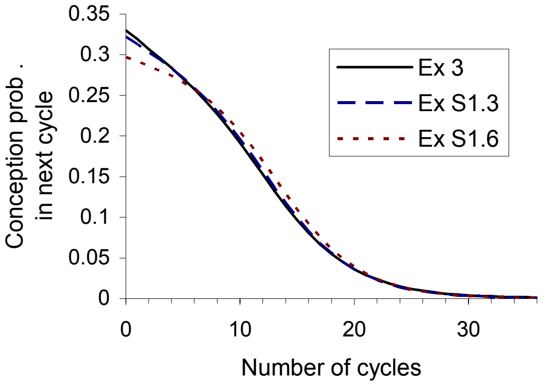

B

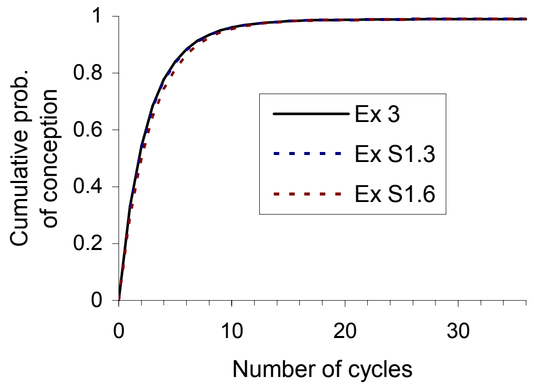

Figure S1.4. This shows, for examples 3, S1.3 and S1.6: (A) probability of conception, conditional on not having already conceived, as a function of the number of elapsed cycles; (B) cumulative conception probability, as a function of number of elapsed cycles.

	Median intrinsic conception rate < 0.05	90th percentile of intrinsic conception rate < 0.2	Probability of conceiving in next cycle < 0.1	Probability of conceiving in next cycle < 0.05	Probability of conceiving in next 12 cycles < 0.5
Example 1	10	11	9	14	8
Example S1.1	9	12	9	13	8
Example S1.4	9	11	8	13	8

Table S1.7: Comparison of examples 1, S1.1 and S1.4: number of cycles of non-conception which must elapse for various metrics of infertility or subfertility to be reached.

	Median intrinsic conception rate < 0.05	90th percentile of intrinsic conception rate < 0.2	Probability of conceiving in next cycle < 0.1	Probability of conceiving in next cycle < 0.05	Probability of conceiving in next 12 cycles < 0.5
Example 2	12	10	8	15	9
Example S1.2	13	13	10	17	11
Example S1.5	11	10	8	15	9

Table S1.8: Comparison of examples 2, S1.2 and S1.5: number of cycles of non-conception which must elapse for various metrics of infertility or subfertility to be reached.

	Median intrinsic conception rate < 0.05	90th percentile of intrinsic conception rate < 0.2	Probability of conceiving in next cycle < 0.1	Probability of conceiving in next cycle < 0.05	Probability of conceiving in next 12 cycles < 0.5
Example 3	14	20	15	19	14
Example S1.3	14	21	15	19	14
Example S1.6	15	21	16	19	14

Table S1.9: Comparison of examples 3, S1.3 and S1.6: number of cycles of non-conception which must elapse for various metrics of infertility or subfertility to be reached.

Appendix C
Methods

Derivation of the formula for updating the distribution of the intrinsic conception rate

As in the main text, let $f(y)$ be the initial (prior) distribution of the intrinsic conception rate for a couple who are trying to conceive. This applies to some population, or to a couple with a given set of objective characteristics. Let $f_n(y)$ be the distribution conditional on n cycles of non-conception. So, for example, $f_3(y)$ is the distribution conditional on three cycles of non-conception. Note that we can write $f(y) = f_0(y)$, i.e. the prior distribution is the distribution conditional on zero cycles of non-conception.

Let X_n denote the event that the couple have not conceived within n cycles. Applying Bayes' theorem for a continuous distribution as given in Lindley [30], the distribution of y conditional on X_n is given by

$$f_n(y) = f(y \mid X_n) = \frac{f(y)p(X_n \mid y)}{\int_0^1 f(y)p(X_n \mid y)dy} \qquad \text{(C1)}$$

In this expression, $p(X_n|y)$ denotes the probability of non-conception within n cycles for a given value of y. This is given by 1 - $P(y,n)$, where $P(y,n)$ is given in equation (2) of the main text. Substituting from equation (2) into (C1) yields

$$f_n(y) = \frac{f(y)(1-y)^n}{\int_0^1 f(y)(1-y)^n dy} \qquad \text{(C2)}$$

Substituting from equation (3) into (C2) yields

$$f_n(y) = \frac{f(y)(1-y)^n}{s(n)}$$

where, as stated in the main text, $s(n)$ is the probability that a couple whose intrinsic conception rate is drawn at random from the distribution $f(y)$ will not conceive within n cycles. We give this result in the main text as equation (4).

It is also useful to have an expression for the probability that a couple drawn from some distribution of the intrinsic conception rate (which is not necessarily the prior distribution) will conceive within m months. Suppose the couple have had n cycles of non-conception where $n \geq 0$. Then the conditional distribution for the couple's intrinsic conception rate is $f_n(y)$. (Note that if $n = 0$ this reduces to $f_0(y)$, which is the same as the prior distribution $f(y)$.) The probability that such a couple will conceive within the next m cycles is given by

$$\int_0^1 f_n(y)(1-y)^m dy \qquad \text{(C3)}$$

Computational method

A program has been written in C under the unix operating system. Given any prior distribution for the intrinsic conception rate, it will numerically compute the following:

- the probability that a couple drawn at random from the prior distribution will have failed to conceive within any specified number of cycles. This is $s(n)$;

- the posterior distribution of the intrinsic conception rate conditional on any specified number of cycles of non-conception. This is $f_n(y)$;

- any specified percentiles of the distribution $f_n(y)$;

- the probability that a couple drawn at random from the distribution $f_n(y)$ will conceive within a specified number *m* of cycles. This is therefore the probability that a couple who have failed to conceive within *n* cycles will conceive within *m* additional cycles.

The probability distribution of the intrinsic conception rate is stored in the following form: a number p_0 specifies the proportion of the distribution which corresponds to sterility ($y = 0$), and the remainder of the distribution, i.e. the continuous part, is stored as an array of 10001 numbers. These numbers represent values of *y* from 0.0 to 1.0 inclusive, in steps of 0.0001.

The program begins with the following inputs:

- the proportion of the prior distribution which is initially sterile. We can label this $p_0(0)$.

- the relative probability densities for the remainder of the distribution, as an array of 10001 numbers corresponding to all values of *y* from 0.0 to 1.0 in steps of 0.0001. We can label this array $f_{REL}[i]$, where $0 \le i \le 10000$. Normalisation is automatically performed by the program (see below).

The important functions in the program are as follows:

Normalisation. From the relative probability densities $f_{REL}[i]$ in the input, the program calculates initial probability densities as an array $f_0[i]$, where *i* is an index number from 0 to 10000 and the corresponding value of *y* is equal to $0.0001 \times i$. The values for the array $f_0[i]$ are obtained by multiplying $f_{REL}[i]$ by a *normalisation constant*. The normalisation constant is calculated so as to ensure that the total probability represented by the continuous part of the distribution is $1 - p_0(0)$.

Computation of a posterior distribution. The prior distribution of the intrinsic conception rate is stored as an array f_0[i], where $0 \le i \le 10000$, and an initial proportion that are sterile $p_0(0)$. From these inputs the program computes the posterior distribution of the intrinsic conception rate conditional on *n* cycles of non-conception. This involves computing a new proportion $p_0(n)$ corresponding to sterility and a new array $f_n[i]$ representing the continuous part of the distribution, by numerically applying expression (4) in the main text. This function also returns the probability of not conceiving within *n* cycles.

Probability of conception within m cycles. This is computed for a couple drawn from some general distribution of the intrinsic conception rate. The distribution used as an input to this function is the distribution conditional on *n* cycles of non-conception where $n \ge 0$ (and $n = 0$ corresponds to the prior distribution). The specific inputs are the proportion $p_0(n)$ of the distribution corresponding to sterility and the array $f_n[i]$, where $0 \le i \le 10000$, corresponding to the continuous part of the distribution. This function numerically evaluates expression (C3).

Methods for example 5

Example 5 differs from the other examples in that couples are subject to a declining intrinsic conception rate during the period of attempted conception.

The model is adapted from Leridon [24]. Considering first a single couple, the couple's intrinsic conception rate is assumed to decline linearly over 12.5 years until sterility occurs; before this decline begins, it is at a constant (peak) value. There is variation between couples: they vary in (i) their peak intrinsic conception rate, and (ii) the age at which sterility occurs. The distribution of peak intrinsic conception rate among couples is assumed to be a beta distribution with $\alpha = 3$ and $\beta = 10$ (see Leridon & Slama [34]). The distribution among couples of the female age at which

sterility occurs in given in Leridon [24], Table 2. Peak intrinsic conception rate and age of sterility are assumed to be independent.

The population is divided into 409 cohorts, according to the female age in months at which sterility occurs. The first cohort is already sterile at a female age of 25 years. The last cohort becomes sterile at a female age of 59 years. More generally, the nth cohort becomes sterile at a female age of 25 years + (n – 1) months. (So, for example, the 100th cohort becomes sterile at a female age of 25 years + 99 months, i.e. 33 years and 3 months.) Table 2 in Leridon [24] gives the proportion of the population that are sterile as a function of completed female years. The proportions that become sterile on a monthly basis are estimated using linear interpolation within each year. For example, Leridon [24], Table 2, gives the proportion of couples who are sterile at ages 25 and 26 at 10 and 11 per 1000 respectively. The interpolation we apply means that we assume that 10 + 1/12 per 1000 are sterile at 25 years and one month, 10 + 2/12 per 1000 at 25 years and two months, and so on.

A separate computation is carried out for each cohort, with the distribution of the intrinsic conception rate tracked numerically over successive cycles of attempted conception. The distribution is stored as an array of 10001 numbers, exactly as for examples 1 to 4 (although in those examples the distribution represents the whole population). After each cycle, the distribution is first updated, applying expression [4] in the main text: this calculates the proportion at each value of the intrinsic conception rate that leaves the population because a pregnancy has been achieved. Then, if the cohort is within 12.5 years of the age of sterility, intrinsic conception rates for the cohort as a whole are reduced by the appropriate proportion: this is 1/150 if the cohort goes from 150 to 149 months to sterility (i.e. the completed month takes the cohort to 149 months before sterility), 1/149 if the cohort goes from 149 to 148 months before sterility, and so on. This involves computationally compressing the distribution. For those cohorts which are below their peak fertility but not yet sterile at the start of the attempted conception process, intrinsic conception rates are appropriately reduced before the start of the computational process tracking a cohort over the duration of attempted conception. Some of these latter cohorts reach their age of sterility and therefore become sterile during the period that they are tracked.

A population with a given female age comprises a mixture of all the cohorts. Conception characteristics for such a population are obtained by appropriately combining the results from all cohorts. The population distribution of the intrinsic conception rate for a given number of cycles of non-conception is given by a superposition (i.e. a weighted sum) of the distributions for all the cohorts. The probability of conception in the next month, or more generally the next m months, is given by a weighted mean of the values of this probability over all cohorts. The weight applied to each cohort is given by the proportion of the population that constitutes that cohort.

We have also implemented a modified version of the program, in which reproductive ageing during the period that couples are attempting to conceive is switched off. The purpose was to explore the difference that reproductive ageing during the conception period makes to the calculated number of cycles of attempted conception before different metrics of subfertility are reached (see Table 12 in the main text).

Appendix D: Full tables of fertility metrics

These are fuller versions of tables giving fertility metrics. They give metrics for all values of number of months elapsed from 0 to 24, and include as an additional metric the probability of conception in the next 24 cycles. The tables concerned are: Tables 2 to 5 and 7 to 10 in the main text, for examples 1 to 5, and Tables S1.1 to Table S1.6 for the additional examples S1.1 to S1.6.

Table 2: Fertility metrics as a function of the number of cycles of attempted conception for example 1 (full table)

Number of cycles elapsed	Proportion of remaining population who are sterile	Median intrinsic conception rate in remaining population	90th percentile of intrinsic conception rate in remaining population	Probability of conceiving in next cycle	Probability of conceiving in next 12 cycles	Probability of conceiving in next 24 cycles	Cumulative probability of conception
0	0.050	0.356	0.633	0.364	0.906	0.938	0.000
1	0.079	0.292	0.551	0.303	0.861	0.903	0.364
2	0.113	0.244	0.485	0.255	0.811	0.863	0.557
3	0.151	0.205	0.431	0.217	0.758	0.819	0.670
4	0.193	0.173	0.386	0.186	0.703	0.771	0.741
5	0.237	0.146	0.347	0.159	0.649	0.722	0.789
6	0.282	0.122	0.314	0.138	0.596	0.672	0.823
7	0.327	0.101	0.285	0.119	0.545	0.623	0.847
8	0.372	0.081	0.260	0.103	0.497	0.576	0.865
9	0.414	0.062	0.237	0.090	0.452	0.531	0.879
10	0.455	0.043	0.217	0.078	0.410	0.488	0.890
11	0.494	0.015	0.199	0.068	0.372	0.448	0.899
12	0.530	0.000	0.183	0.060	0.337	0.411	0.906
13	0.564	0.000	0.168	0.053	0.306	0.377	0.911
14	0.596	0.000	0.155	0.047	0.277	0.345	0.916
15	0.625	0.000	0.143	0.041	0.252	0.317	0.920
16	0.651	0.000	0.131	0.036	0.229	0.291	0.923
17	0.676	0.000	0.121	0.032	0.208	0.267	0.926
18	0.699	0.000	0.112	0.029	0.189	0.245	0.928
19	0.719	0.000	0.103	0.026	0.173	0.225	0.931
20	0.739	0.000	0.095	0.023	0.157	0.208	0.932
21	0.756	0.000	0.087	0.021	0.144	0.191	0.934
22	0.772	0.000	0.080	0.019	0.132	0.177	0.935
23	0.787	0.000	0.074	0.017	0.121	0.163	0.936
24	0.800	0.000	0.067	0.015	0.111	0.151	0.938
25	0.813	0.000	0.061	0.014	0.102	0.140	0.938
26	0.824	0.000	0.056	0.013	0.094	0.130	0.939
27	0.835	0.000	0.051	0.012	0.087	0.121	0.940
28	0.844	0.000	0.046	0.011	0.080	0.112	0.941
29	0.853	0.000	0.041	0.010	0.074	0.104	0.941
30	0.862	0.000	0.036	0.009	0.069	0.097	0.942
31	0.869	0.000	0.032	0.008	0.064	0.091	0.942
32	0.877	0.000	0.027	0.007	0.059	0.085	0.943
33	0.883	0.000	0.023	0.007	0.055	0.080	0.943
34	0.889	0.000	0.018	0.006	0.052	0.075	0.944
35	0.895	0.000	0.012	0.006	0.048	0.070	0.944
36	0.900	0.000	0.000	0.005	0.045	0.066	0.944

Table 3: Fertility metrics as a function of the number of cycles of attempted conception for example 2 (full table)

Number of cycles elapsed	Proportion of remaining population who are sterile	Median fertility in remaining population	90th percentile of fertility in remaining population	Probability of conceiving in next cycle	Probability of conceiving in next 12 cycles	Probability of conceiving in next 24 cycles	Cumulative probability of conception
0	0.050	0.264	0.539	0.285	0.856	0.915	0.000
1	0.070	0.218	0.467	0.239	0.812	0.884	0.285
2	0.092	0.183	0.411	0.204	0.768	0.851	0.456
3	0.116	0.157	0.365	0.177	0.724	0.817	0.567
4	0.140	0.135	0.328	0.155	0.682	0.782	0.644
5	0.166	0.118	0.296	0.136	0.641	0.747	0.699
6	0.192	0.103	0.270	0.121	0.601	0.712	0.740
7	0.219	0.090	0.247	0.108	0.564	0.678	0.771
8	0.245	0.079	0.227	0.097	0.529	0.644	0.796
9	0.272	0.069	0.210	0.087	0.495	0.612	0.816
10	0.298	0.061	0.195	0.079	0.464	0.580	0.832
11	0.323	0.053	0.181	0.072	0.435	0.550	0.845
12	0.348	0.046	0.169	0.065	0.407	0.522	0.856
13	0.372	0.039	0.158	0.059	0.381	0.494	0.866
14	0.396	0.033	0.148	0.054	0.358	0.468	0.874
15	0.419	0.027	0.138	0.050	0.335	0.443	0.881
16	0.441	0.022	0.130	0.046	0.315	0.420	0.887
17	0.462	0.016	0.123	0.042	0.295	0.398	0.892
18	0.482	0.010	0.116	0.039	0.277	0.377	0.896
19	0.502	0.000	0.109	0.036	0.261	0.357	0.900
20	0.520	0.000	0.103	0.033	0.245	0.339	0.904
21	0.538	0.000	0.098	0.031	0.231	0.321	0.907
22	0.555	0.000	0.092	0.029	0.217	0.305	0.910
23	0.572	0.000	0.088	0.027	0.205	0.290	0.913
24	0.587	0.000	0.083	0.025	0.193	0.275	0.915
25	0.602	0.000	0.079	0.023	0.182	0.261	0.917
26	0.616	0.000	0.075	0.022	0.172	0.248	0.919
27	0.630	0.000	0.071	0.020	0.163	0.236	0.921
28	0.643	0.000	0.068	0.019	0.154	0.225	0.922
29	0.655	0.000	0.065	0.018	0.146	0.214	0.924
30	0.667	0.000	0.062	0.017	0.138	0.204	0.925
31	0.678	0.000	0.059	0.016	0.131	0.194	0.926
32	0.689	0.000	0.056	0.015	0.124	0.185	0.927
33	0.700	0.000	0.053	0.014	0.118	0.177	0.929
34	0.709	0.000	0.051	0.013	0.112	0.169	0.930
35	0.719	0.000	0.049	0.012	0.107	0.161	0.930
36	0.728	0.000	0.046	0.012	0.101	0.154	0.931

Table 4: Fertility metrics as a function of the number of cycles of attempted conception for example 3 (full table)

Number of cycles elapsed	Proportion of remaining population who are sterile	Median fertility in remaining population	90th percentile of fertility in remaining population	Probability of conceiving in next cycle	Probability of conceiving in next 12 cycles	Probability of conceiving in next 24 cycles	Cumulative probability of conception
0	0.010	0.329	0.440	0.330	0.973	0.989	0.000
1	0.015	0.319	0.427	0.319	0.966	0.984	0.330
2	0.022	0.308	0.415	0.307	0.957	0.977	0.544
3	0.032	0.299	0.404	0.296	0.945	0.967	0.684
4	0.045	0.289	0.393	0.284	0.930	0.953	0.777
5	0.063	0.280	0.383	0.271	0.910	0.935	0.841
6	0.086	0.270	0.372	0.258	0.884	0.912	0.884
7	0.116	0.260	0.362	0.243	0.853	0.882	0.914
8	0.153	0.249	0.351	0.227	0.814	0.844	0.935
9	0.198	0.237	0.341	0.210	0.768	0.799	0.950
10	0.251	0.224	0.330	0.192	0.716	0.746	0.960
11	0.310	0.209	0.319	0.172	0.656	0.686	0.968
12	0.375	0.190	0.308	0.153	0.593	0.622	0.973
13	0.443	0.163	0.296	0.133	0.527	0.554	0.977
14	0.511	0.000	0.284	0.114	0.460	0.486	0.980
15	0.577	0.000	0.271	0.097	0.397	0.420	0.983
16	0.639	0.000	0.257	0.081	0.337	0.358	0.984
17	0.695	0.000	0.243	0.067	0.284	0.302	0.986
18	0.745	0.000	0.228	0.055	0.236	0.253	0.987
19	0.789	0.000	0.212	0.045	0.195	0.209	0.987
20	0.825	0.000	0.194	0.036	0.160	0.173	0.988
21	0.856	0.000	0.173	0.029	0.131	0.142	0.988
22	0.882	0.000	0.145	0.024	0.107	0.116	0.989
23	0.904	0.000	0.000	0.019	0.087	0.095	0.989
24	0.921	0.000	0.000	0.015	0.071	0.078	0.989
25	0.935	0.000	0.000	0.012	0.058	0.064	0.989
26	0.947	0.000	0.000	0.010	0.048	0.052	0.989
27	0.956	0.000	0.000	0.008	0.039	0.043	0.990
28	0.964	0.000	0.000	0.006	0.032	0.035	0.990
29	0.970	0.000	0.000	0.005	0.026	0.029	0.990
30	0.975	0.000	0.000	0.004	0.022	0.024	0.990
31	0.980	0.000	0.000	0.003	0.018	0.020	0.990
32	0.983	0.000	0.000	0.003	0.015	0.016	0.990
33	0.986	0.000	0.000	0.002	0.012	0.014	0.990
34	0.988	0.000	0.000	0.002	0.010	0.011	0.990
35	0.990	0.000	0.000	0.002	0.008	0.010	0.990
36	0.992	0.000	0.000	0.001	0.007	0.008	0.990

Table 5: Fertility metrics as a function of the number of cycles of attempted conception for example 4 (full table)

Number of cycles elapsed	Proportion of remaining population who are sterile	Median fertility in remaining population	90th percentile of fertility in remaining population	Probability of conceiving in next cycle	Probability of conceiving in next 12 cycles	Probability of conceiving in next 24 cycles	Cumulative probability of conception
0	0.050	0.312	0.432	0.288	0.879	0.915	0.000
1	0.070	0.294	0.416	0.264	0.839	0.883	0.288
2	0.095	0.274	0.401	0.238	0.791	0.844	0.476
3	0.125	0.250	0.385	0.212	0.735	0.798	0.601
4	0.159	0.217	0.368	0.185	0.675	0.747	0.686
5	0.195	0.125	0.350	0.159	0.614	0.694	0.744
6	0.232	0.062	0.332	0.135	0.553	0.641	0.785
7	0.269	0.049	0.313	0.114	0.496	0.591	0.814
8	0.303	0.041	0.292	0.096	0.444	0.544	0.835
9	0.335	0.036	0.270	0.080	0.398	0.502	0.851
10	0.365	0.031	0.246	0.068	0.359	0.465	0.863
11	0.391	0.028	0.218	0.057	0.325	0.432	0.872
12	0.415	0.024	0.181	0.049	0.297	0.404	0.879
13	0.436	0.021	0.102	0.043	0.273	0.380	0.885
14	0.456	0.019	0.080	0.038	0.253	0.359	0.890
15	0.474	0.015	0.072	0.033	0.236	0.340	0.894
16	0.490	0.011	0.067	0.030	0.222	0.324	0.898
17	0.505	0.000	0.063	0.027	0.209	0.310	0.901
18	0.519	0.000	0.061	0.025	0.198	0.297	0.904
19	0.532	0.000	0.058	0.023	0.188	0.285	0.906
20	0.545	0.000	0.056	0.022	0.180	0.274	0.908
21	0.557	0.000	0.055	0.020	0.172	0.264	0.910
22	0.569	0.000	0.053	0.019	0.165	0.255	0.912
23	0.580	0.000	0.052	0.018	0.158	0.246	0.914
24	0.590	0.000	0.051	0.017	0.152	0.238	0.915
25	0.600	0.000	0.050	0.016	0.147	0.230	0.917
26	0.610	0.000	0.049	0.016	0.142	0.223	0.918
27	0.620	0.000	0.048	0.015	0.137	0.216	0.919
28	0.629	0.000	0.046	0.014	0.132	0.209	0.921
29	0.639	0.000	0.046	0.014	0.127	0.203	0.922
30	0.647	0.000	0.045	0.013	0.123	0.196	0.923
31	0.656	0.000	0.044	0.013	0.119	0.190	0.924
32	0.665	0.000	0.043	0.012	0.115	0.184	0.925
33	0.673	0.000	0.042	0.012	0.111	0.179	0.926
34	0.681	0.000	0.041	0.011	0.108	0.173	0.927
35	0.689	0.000	0.040	0.011	0.104	0.168	0.927
36	0.696	0.000	0.040	0.011	0.101	0.163	0.928

Table 7: Fertility metrics as a function of the number of cycles of attempted conception for example 5, with attempted conception beginning at a female age of 25 (full table)

Number of cycles elapsed	Proportion of remaining population who are sterile	Median fertility in remaining population	90th percentile of fertility in remaining population	Probability of conceiving in next cycle	Probability of conceiving in next 12 cycles	Probability of conceiving in next 24 cycles	Cumulative probability of conception
0	0.010	0.210	0.380	0.223	0.872	0.952	0.000
1	0.013	0.193	0.354	0.206	0.853	0.942	0.223
2	0.016	0.179	0.331	0.191	0.832	0.930	0.383
3	0.021	0.166	0.311	0.178	0.812	0.918	0.501
4	0.025	0.155	0.293	0.166	0.791	0.905	0.590
5	0.030	0.145	0.276	0.156	0.771	0.891	0.658
6	0.036	0.136	0.262	0.146	0.750	0.877	0.712
7	0.043	0.128	0.248	0.138	0.729	0.861	0.754
8	0.050	0.120	0.236	0.130	0.708	0.845	0.788
9	0.058	0.113	0.225	0.122	0.687	0.829	0.815
10	0.067	0.107	0.214	0.116	0.667	0.811	0.838
11	0.076	0.101	0.205	0.109	0.646	0.794	0.857
12	0.086	0.096	0.196	0.103	0.626	0.775	0.872
13	0.097	0.091	0.188	0.098	0.605	0.757	0.885
14	0.108	0.086	0.180	0.093	0.585	0.738	0.897
15	0.120	0.081	0.173	0.088	0.565	0.719	0.906
16	0.132	0.077	0.166	0.083	0.546	0.699	0.915
17	0.146	0.073	0.159	0.079	0.526	0.679	0.922
18	0.159	0.069	0.153	0.075	0.507	0.660	0.928
19	0.173	0.065	0.148	0.071	0.488	0.640	0.933
20	0.188	0.061	0.142	0.068	0.470	0.620	0.938
21	0.203	0.058	0.137	0.064	0.452	0.600	0.942
22	0.218	0.054	0.132	0.061	0.434	0.580	0.946
23	0.234	0.051	0.127	0.058	0.417	0.561	0.949
24	0.249	0.048	0.123	0.055	0.400	0.542	0.952
25	0.267	0.045	0.119	0.052	0.384	0.522	0.955
26	0.285	0.042	0.114	0.049	0.368	0.504	0.957
27	0.303	0.039	0.110	0.047	0.353	0.485	0.959
28	0.322	0.036	0.107	0.044	0.338	0.467	0.961
29	0.340	0.033	0.103	0.042	0.323	0.449	0.963
30	0.358	0.029	0.099	0.040	0.309	0.432	0.964
31	0.377	0.026	0.096	0.038	0.296	0.415	0.966
32	0.395	0.023	0.093	0.036	0.283	0.399	0.967
33	0.414	0.02	0.089	0.034	0.270	0.383	0.968
34	0.432	0.017	0.086	0.032	0.258	0.367	0.969
35	0.450	0.013	0.083	0.031	0.247	0.352	0.970
36	0.467	0.009	0.080	0.029	0.235	0.338	0.971

Table 8: Fertility metrics as a function of the number of cycles of attempted conception for example 5, with attempted conception beginning at a female age of 30 (full table)

Number of cycles elapsed	Proportion of remaining population who are sterile	Median fertility in remaining population	90th percentile of fertility in remaining population	Probability of conceiving in next cycle	Probability of conceiving in next 12 cycles	Probability of conceiving in next 24 cycles	Cumulative probability of conception
0	0.020	0.195	0.367	0.209	0.839	0.925	0.000
1	0.026	0.178	0.341	0.191	0.814	0.909	0.209
2	0.032	0.164	0.317	0.176	0.789	0.891	0.360
3	0.040	0.151	0.297	0.163	0.763	0.873	0.473
4	0.048	0.139	0.278	0.150	0.737	0.853	0.559
5	0.058	0.129	0.261	0.140	0.711	0.832	0.625
6	0.068	0.119	0.246	0.130	0.685	0.810	0.677
7	0.080	0.111	0.233	0.121	0.658	0.787	0.719
8	0.092	0.103	0.220	0.113	0.632	0.764	0.753
9	0.105	0.096	0.208	0.105	0.607	0.741	0.781
10	0.119	0.089	0.198	0.098	0.581	0.716	0.804
11	0.134	0.083	0.188	0.092	0.556	0.692	0.823
12	0.149	0.077	0.179	0.086	0.532	0.667	0.839
13	0.166	0.072	0.170	0.080	0.508	0.643	0.853
14	0.184	0.066	0.162	0.075	0.485	0.618	0.865
15	0.202	0.061	0.154	0.070	0.462	0.594	0.875
16	0.220	0.057	0.147	0.066	0.440	0.570	0.884
17	0.239	0.052	0.141	0.062	0.418	0.546	0.892
18	0.259	0.048	0.134	0.058	0.398	0.523	0.898
19	0.278	0.044	0.128	0.054	0.378	0.500	0.904
20	0.298	0.040	0.122	0.051	0.359	0.478	0.909
21	0.319	0.036	0.117	0.047	0.340	0.456	0.914
22	0.339	0.032	0.112	0.044	0.323	0.435	0.918
23	0.359	0.028	0.107	0.042	0.306	0.414	0.922
24	0.379	0.024	0.102	0.039	0.290	0.395	0.925
25	0.400	0.021	0.098	0.037	0.274	0.376	0.928
26	0.420	0.017	0.093	0.034	0.259	0.357	0.930
27	0.441	0.013	0.089	0.032	0.245	0.340	0.933
28	0.461	0.009	0.085	0.030	0.232	0.323	0.935
29	0.481	0.005	0.081	0.028	0.219	0.307	0.937
30	0.500	0.000	0.078	0.026	0.208	0.291	0.939
31	0.519	0.000	0.074	0.025	0.196	0.277	0.940
32	0.537	0.000	0.071	0.023	0.185	0.262	0.942
33	0.555	0.000	0.068	0.022	0.175	0.249	0.943
34	0.573	0.000	0.065	0.021	0.166	0.236	0.944
35	0.590	0.000	0.061	0.019	0.157	0.224	0.946
36	0.606	0.000	0.059	0.018	0.148	0.213	0.947

Table 9: Fertility metrics as a function of the number of cycles of attempted conception for example 5, with attempted conception beginning at a female age of 35 (full table)

Number of cycles elapsed	Proportion of remaining population who are sterile	Median fertility in remaining population	90th percentile of fertility in remaining population	Probability of conceiving in next cycle	Probability of conceiving in next 12 cycles	Probability of conceiving in next 24 cycles	Cumulative probability of conception
0	0.051	0.147	0.310	0.162	0.733	0.836	0.000
1	0.062	0.133	0.286	0.147	0.700	0.809	0.162
2	0.074	0.120	0.264	0.134	0.667	0.781	0.285
3	0.088	0.109	0.245	0.122	0.635	0.753	0.381
4	0.102	0.099	0.229	0.112	0.604	0.724	0.456
5	0.117	0.090	0.213	0.102	0.573	0.695	0.517
6	0.133	0.082	0.200	0.094	0.544	0.666	0.566
7	0.149	0.075	0.187	0.087	0.515	0.637	0.607
8	0.166	0.068	0.176	0.080	0.487	0.608	0.641
9	0.184	0.062	0.166	0.074	0.460	0.580	0.670
10	0.202	0.056	0.156	0.068	0.435	0.553	0.694
11	0.220	0.051	0.147	0.063	0.410	0.526	0.715
12	0.239	0.046	0.139	0.058	0.387	0.499	0.733
13	0.260	0.041	0.131	0.054	0.364	0.474	0.749
14	0.280	0.037	0.124	0.050	0.343	0.449	0.762
15	0.301	0.033	0.117	0.046	0.323	0.426	0.774
16	0.323	0.029	0.111	0.043	0.304	0.403	0.785
17	0.344	0.025	0.105	0.040	0.286	0.381	0.794
18	0.365	0.021	0.100	0.037	0.268	0.360	0.802
19	0.386	0.018	0.095	0.034	0.252	0.340	0.809
20	0.406	0.015	0.090	0.032	0.237	0.321	0.816
21	0.427	0.011	0.085	0.030	0.222	0.303	0.822
22	0.447	0.008	0.081	0.028	0.209	0.285	0.827
23	0.467	0.005	0.076	0.026	0.196	0.269	0.832
24	0.486	0.002	0.072	0.024	0.184	0.253	0.836
25	0.507	0.000	0.069	0.022	0.172	0.239	0.840
26	0.528	0.000	0.065	0.021	0.162	0.225	0.844
27	0.548	0.000	0.061	0.019	0.152	0.212	0.847
28	0.567	0.000	0.058	0.018	0.142	0.199	0.850
29	0.586	0.000	0.055	0.017	0.133	0.187	0.853
30	0.604	0.000	0.052	0.016	0.125	0.176	0.855
31	0.622	0.000	0.049	0.015	0.117	0.166	0.857
32	0.639	0.000	0.046	0.014	0.110	0.156	0.860
33	0.655	0.000	0.044	0.013	0.103	0.147	0.861
34	0.671	0.000	0.041	0.012	0.097	0.138	0.863
35	0.686	0.000	0.039	0.011	0.091	0.130	0.865
36	0.700	0.000	0.036	0.010	0.085	0.122	0.866

Table 10: Fertility metrics as a function of the number of cycles of attempted conception for example 5, with attempted conception beginning at a female age of 40 (full table)

Number of cycles elapsed	Proportion of remaining population who are sterile	Median fertility in remaining population	90th percentile of fertility in remaining population	Probability of conceiving in next cycle	Probability of conceiving in next 12 cycles	Probability of conceiving in next 24 cycles	Cumulative probability of conception
0	0.166	0.070	0.194	0.087	0.494	0.607	0.000
1	0.185	0.063	0.177	0.079	0.463	0.575	0.087
2	0.205	0.057	0.163	0.071	0.434	0.544	0.159
3	0.225	0.051	0.151	0.065	0.406	0.514	0.219
4	0.244	0.046	0.141	0.059	0.380	0.485	0.269
5	0.264	0.041	0.131	0.054	0.356	0.458	0.313
6	0.284	0.037	0.123	0.050	0.333	0.431	0.350
7	0.304	0.033	0.115	0.046	0.312	0.406	0.382
8	0.324	0.029	0.108	0.042	0.292	0.382	0.411
9	0.344	0.025	0.101	0.039	0.273	0.359	0.435
10	0.364	0.022	0.095	0.036	0.255	0.338	0.457
11	0.383	0.019	0.090	0.033	0.238	0.317	0.477
12	0.402	0.015	0.085	0.031	0.223	0.297	0.494
13	0.422	0.012	0.080	0.028	0.208	0.279	0.510
14	0.441	0.009	0.075	0.026	0.194	0.261	0.523
15	0.460	0.007	0.071	0.024	0.181	0.244	0.536
16	0.479	0.004	0.067	0.022	0.169	0.228	0.547
17	0.498	0.001	0.063	0.021	0.158	0.213	0.557
18	0.516	0.000	0.060	0.019	0.147	0.199	0.567
19	0.533	0.000	0.056	0.018	0.137	0.186	0.575
20	0.550	0.000	0.053	0.017	0.128	0.174	0.583
21	0.567	0.000	0.050	0.015	0.119	0.162	0.589
22	0.583	0.000	0.047	0.014	0.111	0.151	0.596
23	0.599	0.000	0.044	0.013	0.103	0.140	0.601
24	0.614	0.000	0.042	0.012	0.096	0.130	0.607
25	0.630	0.000	0.039	0.011	0.089	0.121	0.612
26	0.646	0.000	0.037	0.011	0.083	0.112	0.616
27	0.661	0.000	0.034	0.010	0.077	0.104	0.620
28	0.676	0.000	0.032	0.009	0.071	0.097	0.624
29	0.690	0.000	0.030	0.008	0.066	0.090	0.627
30	0.703	0.000	0.028	0.008	0.061	0.083	0.630
31	0.716	0.000	0.026	0.007	0.057	0.077	0.633
32	0.728	0.000	0.024	0.007	0.052	0.071	0.636
33	0.740	0.000	0.022	0.006	0.049	0.065	0.638
34	0.751	0.000	0.020	0.006	0.045	0.060	0.641
35	0.762	0.000	0.019	0.005	0.041	0.055	0.643
36	0.772	0.000	0.017	0.005	0.038	0.051	0.644

Table S1.1: Fertility metrics as a function of the number of cycles of attempted conception for example S1.1 (full table)

Number of cycles elapsed	Proportion of remaining population who are sterile	Median intrinsic conception rate in remaining population	90th percentile of intrinsic conception rate in remaining population	Probability of conceiving in next cycle	Probability of conceiving in next 12 cycles	Probability of conceiving in next 24 cycles	Cumulative probability of conception
0	0.050	0.389	0.617	0.380	0.917	0.941	0.000
1	0.081	0.336	0.559	0.327	0.874	0.906	0.380
2	0.120	0.284	0.505	0.279	0.821	0.861	0.583
3	0.166	0.236	0.458	0.237	0.761	0.809	0.699
4	0.218	0.193	0.417	0.200	0.698	0.752	0.771
5	0.273	0.154	0.380	0.169	0.633	0.693	0.817
6	0.328	0.120	0.345	0.142	0.570	0.633	0.848
7	0.382	0.089	0.310	0.119	0.510	0.575	0.869
8	0.434	0.059	0.278	0.101	0.454	0.520	0.885
9	0.483	0.027	0.249	0.085	0.404	0.469	0.896
10	0.528	0.000	0.223	0.072	0.359	0.423	0.905
11	0.568	0.000	0.200	0.061	0.319	0.381	0.912
12	0.606	0.000	0.180	0.052	0.284	0.343	0.917
13	0.639	0.000	0.161	0.045	0.253	0.310	0.922
14	0.669	0.000	0.145	0.039	0.226	0.280	0.925
15	0.696	0.000	0.131	0.034	0.202	0.253	0.928
16	0.720	0.000	0.118	0.029	0.181	0.230	0.931
17	0.742	0.000	0.106	0.026	0.163	0.209	0.933
18	0.762	0.000	0.095	0.023	0.147	0.190	0.934
19	0.780	0.000	0.086	0.020	0.133	0.174	0.936
20	0.796	0.000	0.077	0.018	0.120	0.159	0.937
21	0.810	0.000	0.069	0.016	0.109	0.146	0.938
22	0.823	0.000	0.061	0.014	0.099	0.134	0.939
23	0.835	0.000	0.054	0.013	0.091	0.123	0.940
24	0.846	0.000	0.048	0.011	0.083	0.113	0.941
25	0.855	0.000	0.041	0.010	0.076	0.105	0.942
26	0.864	0.000	0.036	0.009	0.070	0.097	0.942
27	0.872	0.000	0.030	0.009	0.064	0.090	0.943
28	0.880	0.000	0.025	0.008	0.059	0.084	0.943
29	0.887	0.000	0.019	0.007	0.055	0.078	0.944
30	0.893	0.000	0.013	0.006	0.051	0.072	0.944
31	0.899	0.000	0.005	0.006	0.047	0.068	0.944
32	0.904	0.000	0.000	0.005	0.044	0.063	0.945
33	0.909	0.000	0.000	0.005	0.041	0.059	0.945
34	0.914	0.000	0.000	0.005	0.038	0.055	0.945
35	0.918	0.000	0.000	0.004	0.035	0.052	0.946
36	0.922	0.000	0.000	0.004	0.033	0.049	0.946

Table S1.2: Fertility metrics as a function of the number of cycles of attempted conception for example S1.2 (full table)

Number of cycles elapsed	Proportion of remaining population who are sterile	Median intrinsic conception rate in remaining population	90th percentile of intrinsic conception rate in remaining population	Probability of conceiving in next cycle	Probability of conceiving in next 12 cycles	Probability of conceiving in next 24 cycles	Cumulative probability of conception
0	0.050	0.243	0.385	0.237	0.869	0.927	0.000
1	0.066	0.227	0.369	0.221	0.842	0.906	0.237
2	0.084	0.209	0.353	0.204	0.811	0.882	0.406
3	0.106	0.192	0.335	0.188	0.778	0.855	0.527
4	0.130	0.174	0.318	0.172	0.742	0.824	0.616
5	0.157	0.156	0.302	0.157	0.704	0.791	0.682
6	0.186	0.140	0.286	0.143	0.665	0.756	0.732
7	0.218	0.124	0.271	0.130	0.626	0.720	0.770
8	0.250	0.108	0.257	0.117	0.586	0.682	0.800
9	0.283	0.094	0.243	0.106	0.547	0.645	0.823
10	0.317	0.081	0.230	0.095	0.509	0.607	0.842
11	0.350	0.068	0.216	0.086	0.473	0.570	0.857
12	0.383	0.056	0.202	0.077	0.438	0.534	0.869
13	0.415	0.044	0.189	0.070	0.405	0.500	0.880
14	0.446	0.033	0.177	0.063	0.375	0.467	0.888
15	0.476	0.020	0.165	0.056	0.346	0.436	0.895
16	0.504	0.000	0.154	0.051	0.319	0.406	0.901
17	0.532	0.000	0.144	0.046	0.295	0.379	0.906
18	0.557	0.000	0.134	0.042	0.272	0.353	0.910
19	0.581	0.000	0.125	0.038	0.251	0.329	0.914
20	0.604	0.000	0.117	0.034	0.232	0.307	0.917
21	0.625	0.000	0.109	0.031	0.215	0.287	0.920
22	0.645	0.000	0.102	0.028	0.199	0.268	0.923
23	0.664	0.000	0.095	0.026	0.184	0.250	0.925
24	0.682	0.000	0.089	0.024	0.171	0.234	0.927
25	0.698	0.000	0.083	0.022	0.159	0.219	0.928
26	0.713	0.000	0.078	0.020	0.148	0.205	0.930
27	0.728	0.000	0.073	0.018	0.137	0.192	0.931
28	0.741	0.000	0.068	0.017	0.128	0.180	0.933
29	0.754	0.000	0.064	0.015	0.119	0.169	0.934
30	0.765	0.000	0.059	0.014	0.112	0.159	0.935
31	0.776	0.000	0.055	0.013	0.104	0.150	0.936
32	0.787	0.000	0.052	0.012	0.098	0.141	0.936
33	0.797	0.000	0.048	0.011	0.091	0.133	0.937
34	0.806	0.000	0.045	0.011	0.086	0.125	0.938
35	0.814	0.000	0.042	0.010	0.081	0.118	0.939
36	0.822	0.000	0.039	0.009	0.076	0.112	0.939

Table S1.3: Fertility metrics as a function of the number of cycles of attempted conception for example S1.3 (full table)

Number of cycles elapsed	Proportion of remaining population who are sterile	Median intrinsic conception rate in remaining population	90th percentile of intrinsic conception rate in remaining population	Probability of conceiving in next cycle	Probability of conceiving in next 12 cycles	Probability of conceiving in next 24 cycles	Cumulative probability of conception
0	0.010	0.324	0.421	0.322	0.973	0.989	0.000
1	0.015	0.316	0.413	0.313	0.967	0.984	0.322
2	0.021	0.307	0.405	0.303	0.958	0.978	0.534
3	0.031	0.299	0.396	0.294	0.947	0.968	0.675
4	0.044	0.290	0.387	0.283	0.932	0.955	0.771
5	0.061	0.280	0.378	0.272	0.914	0.938	0.836
6	0.083	0.271	0.368	0.259	0.890	0.915	0.880
7	0.113	0.261	0.359	0.246	0.859	0.886	0.911
8	0.149	0.250	0.350	0.230	0.822	0.849	0.933
9	0.194	0.239	0.341	0.214	0.777	0.804	0.948
10	0.247	0.226	0.331	0.196	0.724	0.751	0.959
11	0.307	0.213	0.322	0.177	0.665	0.691	0.967
12	0.373	0.198	0.311	0.157	0.600	0.625	0.973
13	0.442	0.180	0.299	0.137	0.532	0.556	0.977
14	0.513	0.000	0.287	0.118	0.464	0.486	0.980
15	0.581	0.000	0.274	0.100	0.398	0.417	0.983
16	0.646	0.000	0.260	0.083	0.336	0.353	0.985
17	0.704	0.000	0.245	0.068	0.280	0.295	0.986
18	0.756	0.000	0.231	0.056	0.231	0.243	0.987
19	0.800	0.000	0.216	0.045	0.188	0.199	0.988
20	0.838	0.000	0.200	0.036	0.153	0.161	0.988
21	0.869	0.000	0.184	0.029	0.123	0.130	0.988
22	0.895	0.000	0.163	0.023	0.099	0.105	0.989
23	0.916	0.000	0.000	0.018	0.079	0.084	0.989
24	0.933	0.000	0.000	0.014	0.063	0.067	0.989
25	0.946	0.000	0.000	0.011	0.050	0.054	0.989
26	0.957	0.000	0.000	0.009	0.040	0.043	0.990
27	0.966	0.000	0.000	0.007	0.032	0.034	0.990
28	0.972	0.000	0.000	0.006	0.026	0.027	0.990
29	0.978	0.000	0.000	0.004	0.020	0.022	0.990
30	0.982	0.000	0.000	0.004	0.016	0.018	0.990
31	0.986	0.000	0.000	0.003	0.013	0.014	0.990
32	0.989	0.000	0.000	0.002	0.010	0.011	0.990
33	0.991	0.000	0.000	0.002	0.008	0.009	0.990
34	0.993	0.000	0.000	0.001	0.007	0.007	0.990
35	0.994	0.000	0.000	0.001	0.005	0.006	0.990
36	0.995	0.000	0.000	0.001	0.004	0.005	0.990

Table S1.4: Fertility metrics as a function of the number of cycles of attempted conception for example S1.4 (full table)

Number of cycles elapsed	Proportion of remaining population who are sterile	Median intrinsic conception rate in remaining population	90th percentile of intrinsic conception rate in remaining population	Probability of conceiving in next cycle	Probability of conceiving in next 12 cycles	Probability of conceiving in next 24 cycles	Cumulative probability of conception
0	0.050	0.388	0.557	0.361	0.913	0.939	0.000
1	0.078	0.335	0.538	0.319	0.871	0.906	0.361
2	0.115	0.281	0.510	0.277	0.820	0.863	0.565
3	0.159	0.231	0.473	0.236	0.761	0.812	0.685
4	0.208	0.187	0.429	0.200	0.698	0.757	0.760
5	0.260	0.149	0.385	0.168	0.634	0.699	0.808
6	0.312	0.116	0.343	0.141	0.572	0.641	0.840
7	0.364	0.088	0.305	0.118	0.514	0.585	0.863
8	0.413	0.062	0.272	0.100	0.461	0.533	0.879
9	0.458	0.038	0.243	0.085	0.413	0.484	0.891
10	0.501	0.000	0.218	0.072	0.369	0.440	0.900
11	0.540	0.000	0.196	0.062	0.331	0.399	0.907
12	0.576	0.000	0.176	0.053	0.297	0.363	0.913
13	0.608	0.000	0.159	0.046	0.266	0.330	0.918
14	0.637	0.000	0.144	0.040	0.240	0.301	0.922
15	0.664	0.000	0.131	0.035	0.216	0.274	0.925
16	0.689	0.000	0.119	0.031	0.195	0.251	0.927
17	0.711	0.000	0.108	0.027	0.177	0.229	0.930
18	0.731	0.000	0.098	0.024	0.161	0.210	0.932
19	0.749	0.000	0.089	0.022	0.146	0.193	0.933
20	0.765	0.000	0.081	0.019	0.133	0.178	0.935
21	0.780	0.000	0.073	0.017	0.122	0.164	0.936
22	0.794	0.000	0.066	0.016	0.112	0.152	0.937
23	0.807	0.000	0.060	0.014	0.102	0.140	0.938
24	0.818	0.000	0.054	0.013	0.094	0.130	0.939
25	0.829	0.000	0.049	0.012	0.087	0.121	0.940
26	0.838	0.000	0.044	0.011	0.080	0.112	0.940
27	0.847	0.000	0.039	0.010	0.074	0.105	0.941
28	0.856	0.000	0.034	0.009	0.069	0.098	0.942
29	0.863	0.000	0.030	0.008	0.064	0.091	0.942
30	0.870	0.000	0.026	0.007	0.059	0.085	0.943
31	0.877	0.000	0.022	0.007	0.055	0.080	0.943
32	0.883	0.000	0.018	0.006	0.052	0.075	0.943
33	0.889	0.000	0.014	0.006	0.048	0.070	0.944
34	0.894	0.000	0.010	0.005	0.045	0.066	0.944
35	0.899	0.000	0.004	0.005	0.042	0.062	0.944
36	0.903	0.000	0.000	0.005	0.040	0.059	0.945

Table S1.5: Fertility metrics as a function of the number of cycles of attempted conception for example S1.5 (full table)

Number of cycles elapsed	Proportion of remaining population who are sterile	Median intrinsic conception rate in remaining population	90th percentile of intrinsic conception rate in remaining population	Probability of conceiving in next cycle	Probability of conceiving in next 12 cycles	Probability of conceiving in next 24 cycles	Cumulative probability of conception
0	0.050	0.263	0.486	0.266	0.845	0.905	0.000
1	0.068	0.219	0.452	0.232	0.801	0.874	0.266
2	0.089	0.183	0.413	0.201	0.756	0.840	0.436
3	0.111	0.153	0.374	0.175	0.710	0.803	0.550
4	0.135	0.129	0.337	0.152	0.665	0.766	0.629
5	0.159	0.109	0.303	0.133	0.621	0.730	0.685
6	0.183	0.093	0.273	0.117	0.581	0.694	0.727
7	0.208	0.080	0.248	0.104	0.542	0.659	0.759
8	0.232	0.069	0.226	0.092	0.507	0.625	0.784
9	0.255	0.060	0.206	0.083	0.474	0.593	0.804
10	0.278	0.052	0.189	0.074	0.443	0.563	0.820
11	0.300	0.045	0.175	0.067	0.415	0.534	0.834
12	0.322	0.039	0.162	0.061	0.389	0.507	0.845
13	0.343	0.033	0.150	0.056	0.365	0.482	0.854
14	0.363	0.029	0.140	0.051	0.343	0.458	0.862
15	0.383	0.024	0.131	0.047	0.322	0.435	0.869
16	0.401	0.020	0.122	0.043	0.303	0.414	0.875
17	0.419	0.017	0.115	0.040	0.286	0.394	0.881
18	0.437	0.013	0.108	0.037	0.269	0.375	0.886
19	0.453	0.010	0.102	0.034	0.254	0.357	0.890
20	0.469	0.007	0.096	0.032	0.240	0.340	0.893
21	0.485	0.004	0.091	0.029	0.227	0.324	0.897
22	0.499	0.000	0.086	0.028	0.215	0.310	0.900
23	0.514	0.000	0.082	0.026	0.204	0.296	0.903
24	0.527	0.000	0.077	0.024	0.194	0.282	0.905
25	0.540	0.000	0.074	0.023	0.184	0.270	0.907
26	0.553	0.000	0.070	0.021	0.175	0.258	0.910
27	0.565	0.000	0.067	0.020	0.166	0.247	0.911
28	0.576	0.000	0.064	0.019	0.158	0.237	0.913
29	0.587	0.000	0.061	0.018	0.151	0.227	0.915
30	0.598	0.000	0.058	0.017	0.144	0.218	0.916
31	0.608	0.000	0.056	0.016	0.137	0.209	0.918
32	0.618	0.000	0.053	0.015	0.131	0.200	0.919
33	0.627	0.000	0.051	0.014	0.125	0.193	0.920
34	0.637	0.000	0.049	0.014	0.120	0.185	0.921
35	0.645	0.000	0.047	0.013	0.115	0.178	0.923
36	0.654	0.000	0.045	0.012	0.110	0.171	0.924

Table S1.6: Fertility metrics as a function of the number of cycles of attempted conception for example S1.6 (full table)

Number of cycles elapsed	Proportion of remaining population who are sterile	Median intrinsic conception rate in remaining population	90th percentile of intrinsic conception rate in remaining population	Probability of conceiving in next cycle	Probability of conceiving in next 12 cycles	Probability of conceiving in next 24 cycles	Cumulative probability of conception
0	0.010	0.299	0.367	0.297	0.971	0.989	0.000
1	0.014	0.295	0.363	0.292	0.965	0.985	0.297
2	0.020	0.291	0.359	0.286	0.958	0.979	0.502
3	0.028	0.286	0.355	0.280	0.949	0.971	0.645
4	0.039	0.282	0.351	0.274	0.937	0.960	0.744
5	0.054	0.277	0.346	0.266	0.921	0.945	0.814
6	0.073	0.272	0.342	0.257	0.901	0.925	0.864
7	0.099	0.266	0.337	0.247	0.875	0.900	0.899
8	0.131	0.260	0.332	0.235	0.842	0.868	0.924
9	0.171	0.252	0.327	0.221	0.802	0.827	0.942
10	0.220	0.244	0.321	0.205	0.754	0.779	0.955
11	0.277	0.234	0.315	0.188	0.698	0.722	0.964
12	0.341	0.221	0.308	0.169	0.635	0.658	0.971
13	0.410	0.202	0.301	0.149	0.567	0.589	0.976
14	0.482	0.165	0.293	0.129	0.497	0.517	0.979
15	0.554	0.000	0.284	0.110	0.427	0.445	0.982
16	0.622	0.000	0.274	0.092	0.361	0.377	0.984
17	0.685	0.000	0.263	0.076	0.300	0.314	0.985
18	0.741	0.000	0.250	0.061	0.246	0.258	0.987
19	0.790	0.000	0.236	0.049	0.199	0.209	0.987
20	0.831	0.000	0.219	0.039	0.160	0.168	0.988
21	0.865	0.000	0.198	0.031	0.128	0.135	0.988
22	0.892	0.000	0.160	0.024	0.101	0.107	0.989
23	0.915	0.000	0.000	0.019	0.080	0.085	0.989
24	0.932	0.000	0.000	0.015	0.063	0.067	0.989
25	0.947	0.000	0.000	0.012	0.050	0.053	0.989
26	0.958	0.000	0.000	0.009	0.039	0.042	0.990
27	0.967	0.000	0.000	0.007	0.031	0.033	0.990
28	0.973	0.000	0.000	0.006	0.025	0.026	0.990
29	0.979	0.000	0.000	0.004	0.020	0.021	0.990
30	0.983	0.000	0.000	0.003	0.015	0.017	0.990
31	0.987	0.000	0.000	0.003	0.012	0.013	0.990
32	0.989	0.000	0.000	0.002	0.010	0.011	0.990
33	0.991	0.000	0.000	0.002	0.008	0.008	0.990
34	0.993	0.000	0.000	0.001	0.006	0.007	0.990
35	0.994	0.000	0.000	0.001	0.005	0.005	0.990
36	0.996	0.000	0.000	0.001	0.004	0.004	0.990
